# Variations in flanking or less conserved positions of Reb1 and Abf1 consensus binding sites lead to major changes in their ability to modulate nucleosome sliding activity

**DOI:** 10.1186/s40659-025-00627-0

**Published:** 2025-07-29

**Authors:** Fernanda Raiqueo, Roberto Amigo, José L. Gutiérrez

**Affiliations:** Departamento de Bioquímica y Biología Molecular, Facultad de Ciencias Biológicas, Universidad de Concepción, Concepción, 4070043 Chile

**Keywords:** Chromatin dynamics, Chromatin remodeling, Nucleosome sliding, Nucleosome remodeling, ISW1a, Reb1, Abf1, TFBS motifs, Residence time, Dwell time

## Abstract

**Background:**

Maintenance of nucleosome-free regions at gene regulatory regions conform a relevant aspect within chromatin dynamics. In the yeast *Saccharomyces cerevisiae*, Reb1 and Abf1 are among the transcriptions factors that perform this molecular function. These factors are thought to act as a barrier to nucleosome sliding that chromatin remodeling complexes such as ISW1a perform towards this region, being binding affinity a critical feature to act as a barrier. In this regard, sequence variations at positions flanking transcription factor binding sites could affect DNA shape features and, in turn, binding strength. In addition, recent studies have shown that positions of low conservation and/or flanking sequences might vary from gene bodies to gene regulatory regions. Considering these issues, we aimed to analyze whether variations in flanking or less conserved positions of Reb1 and Abf1 target sequences affect their binding affinity, especially dwell time, and their ability to hinder ISW1a’s sliding activity.

**Results:**

We found that sequence changes at these positions deeply affect binding strength, particularly dwell time, and the ability to hinder ISW1a’s sliding activity. Importantly, even under conditions where a markedly higher transcription factor concentration for a weak binding site was used to compare it to a strong binding site under an equal binding saturation level, the strong site displayed a significantly higher ability to hinder sliding activity. Moreover, genome-wide analyses showed that the sequence variants of Reb1 and Abf1 binding sites conferring this ability to hinder sliding activity to these factors are enriched at promoter regions relative to gene bodies.

**Conclusions:**

Our findings show that dwell time is a key feature to hinder nucleosome sliding activity. For Reb1 and Abf1 factors, sequence variation at less conserved positions of their binding sites strongly affects this feature. The differential frequency at these positions found at promoter regions, relative to gene bodies, highlights the relevance of including this type of comparison in certain strategies used to determine the consensus binding site for transcription factors. To determine the molecular functions that require long dwell times and the transcription factors responsible for these tasks will significantly contribute to untangle the grammar of *cis*-regulatory elements.

**Supplementary Information:**

The online version contains supplementary material available at 10.1186/s40659-025-00627-0.

## Background

A noteworthy aspect of the chromatin dynamics involved in transcriptional regulation corresponds to generation and maintenance of nucleosome-free regions (NFRs) at gene regulatory regions. Factors concurring with these processes include transcription factors (TFs), ATP-dependent chromatin remodeling complexes (CRCs) and DNA sequences [1, 2]. In this context, it has been observed that defined CRCs play opposite roles at these regions. In the yeast *Saccharomyces cerevisiae*, CRCs such as RSC contribute to establishment and maintenance of NFRs, while other CRCs, including ISW1a, perform NFR shrinking through their nucleosome sliding activity [3–5]. Regarding transcription factors, these may assist nucleosome disassembly and/or contribute to sustain consolidated NFRs [6–9]. In *S. cerevisiae*, transcription factors such as Reb1, Abf1, Rap1 and Cbf1 - termed general regulatory factors (GRFs) - play a critical role within these dynamics. It has been determined that GRFs, once bound to their cognate DNA sequences, can act as a barrier against NFR shrinking mediated by CRCs that attempt to populate NFRs with nucleosomes [5, 8, 10].

A common approach used to define the target sequence of transcription factors is to define their consensus binding site using ChIP-seq binding peaks collected from the whole genome. In this regard, recent findings have focused on key aspects that relate binding site variants to protein-DNA binding affinity. Firstly, a linkage between sequence variants of the canonical binding site of defined GRFs and their preferential occupancy at gene promoters, relative to gene bodies, has been determined [11]. Secondly, it has been shown that sequence variations at positions flanking transcription factor binding sites (TFBSs) could affect DNA shape features and, in turn, binding strength of TFs [12, 13]. These findings suggest that consensus binding sites obtained from the whole genome might not reflect the highest affinity target sequence for a TF and points that positions of low conservation and/or flanking sequences might vary from gene bodies to gene regulatory regions, affecting binding affinity. In this context, several studies point that the strength of GRFs binding to their target sequence, particularly dwell time, deeply affect their function at gene regulatory regions. Indeed, it has been described that residence time is a critical feature for Rap1 to exert its transcriptional activation function [14]. Subsequent studies have shown that this GRF is able to invade and open closed chromatin conformations and then sustaining long residence times once bound to their target sequences on naked DNA [9]. Likewise, among Abf1 binding sites, those harboring long dwell times display the largest NFRs [15]. In addition, Reb1 and Cbf1 are able to sustain low dissociation rates upon binding to their target sequences embedded near the entry-exit site of nucleosomes [16]. In agreement with these findings, we have recently shown that binding strength, particularly dwell time, correlates with the ability to hinder ISW1a’s nucleosome sliding activity. Among the GRFs tested, only Rap1 displayed a long dwell time and ability to hinder ISW1a’s sliding activity, while the Reb1 and Abf1 displayed short dwell times and did not significantly hinder the activity of this complex [17].

In our current study, we aimed to analyze whether variations in flanking or less conserved positions of Reb1 and Abf1 target sequences influence on their binding affinity and their ability to hinder ISW1a’s sliding activity. Our results showed that sequence changes at these positions deeply affect binding strength, dwell time and the ability to hinder the sliding activity of the ISW1a complex. Importantly, strong binding sites displayed a significantly higher ability to hinder ISW1a’s sliding activity, even under conditions where a markedly higher GRF concentration for the weak binding site was used to compare weak to strong binding sites under equal binding saturation levels. Moreover, genome-wide analyses showed that the sequence variants that confer the ability to hinder sliding activity to Reb1 and Abf1 are enriched at promoter regions relative to gene bodies. These findings demonstrate the marked differences that could be displayed by binding site variants that are compatible with a consensus binding site, including molecular functions such as maintenance of NFRs. Our results highlight the relevance of determining the molecular functions that require long residence times, the TFs that exert these functions and the binding properties of these TFs for the full set of their binding site variants.

## Results

We have recently performed a comparative analysis on modulation of ISW1a’s sliding activity by the main *S. cerevisiae* GRFs [17], finding that Rap1 has the property of hindering ISW1a’s sliding activity. We also found that this ability correlates with binding strength, particularly in terms of dwell time. In addition, we found that the binding strength of Rap1 correlates with its ability to maintain low nucleosome occupancy and hinder histone deposition [17]. In light of these findings, we aimed to find whether there are Reb1 and Abf1 binding site variants that could confer binding strengths higher than those found in our previous study and whether these higher binding strengths endow these GRFs with the property of hindering ISW1a’s nucleosome sliding activity.

### Sequence variation at flanking or less conserved positions of Reb1 and Abf1 consensus binding sites involve major differences in dwell time

As detailed in the Introduction, recent findings point to a linkage between defined GRFs’ binding site variants and their preferential occupancy at gene promoters, relative to gene bodies, while other findings show that sequence variations in flanking positions of TFBSs could deeply affect TFs’ binding strength [11–13]. In light of these evidences, we focused our search in subtle variations from the canonical binding sites. These subtle variations were defined in terms of flanking sequences and less conserved positions within Reb1 and Abf1 consensus binding sites. Four different variants were tested for each GRF.

**Fig. 1:**
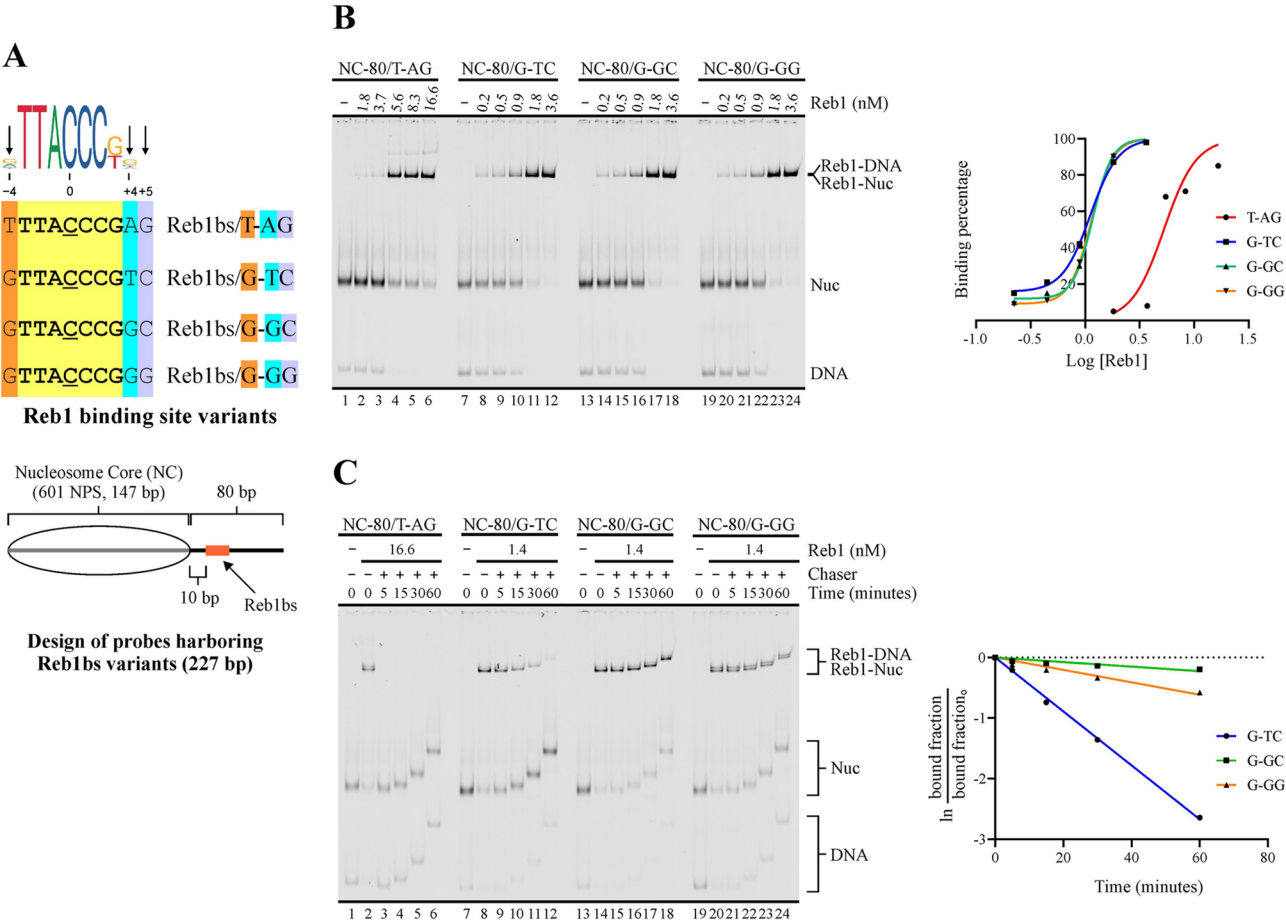
Variations at flanking or less conserved positions of Reb1 consensus binding sites involve major changes in affinity and dwell time. (**A**) *Upper panel*: Depiction of Reb1 binding site variants tested. The name of each variant is based on the specific nucleotides present at positions −4, +4 and +5. Displayed on top is the consensus binding site, according to the JASPAR database [18]. *Lower panel*: Schematic representation of the nucleosome probes used in the assays. 601 NPS = nucleosome positioning region of the 601 sequence (gray bar). The oval represents the translational position adopted by the nucleosome core upon reconstitution, which spans the 601 region. Probe names indicate length of linker downstream (right) of the core and binding site variant located in the linker DNA region. **(B**,** C**) Apparent K_d_ (**B**) and dissociation kinetics (**C**) determinations for Reb1BS variants. The gel images correspond to electrophoresis in a non-denaturing polyacrylamide gel; each one is representative of three independent assays. The probe used in each reaction and Reb1 concentrations are depicted at the top of gel images; migrations of free DNA probe (DNA), nucleosome probe (Nuc), DNA probe bound by Reb1 (Reb1-DNA) and nucleosome probe bound by Reb1 (Reb1-Nuc) are indicated at the right. In addition, for the dissociation kinetics analysis, the different time points and use of an unlabeled double-stranded oligonucleotide harboring a Reb1BS for Reb1 removal (chaser) are depicted at the top of the gel image. The graphs at the right of each gel image correspond to densitometric quantification of binding percentages used to calculate K_d_ (**B**) and K_off_ (**C**) values, which are displayed in Table 1

**Fig. 2:**
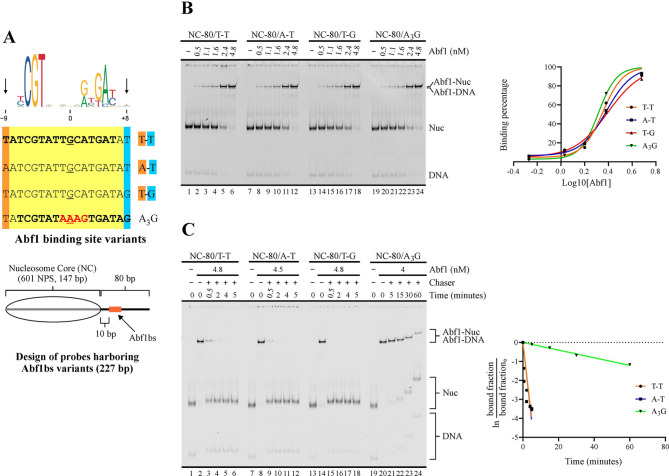
Variations at less conserved positions of Abf1 consensus binding sites involve major changes in dwell time. (**A**) *Upper panel*: Depiction of Abf1 binding site variants tested. The name of each variant is based on the specific nucleotides present at positions −9 and +8 or present at positions −1 to +2 in the case of the A_3_G variant. Displayed on top is the consensus binding site, according to the JASPAR database [18]. *Lower panel*: Schematic representation of the nucleosome probes used in the assays. 601 NPS = nucleosome positioning region of the 601 sequence (gray bar). The oval represents the translational position adopted by the nucleosome core upon reconstitution, which spans the 601 region. Probe names indicate length of linker downstream (right) of the core and binding site variant located in the linker DNA region. **(B**,** C**) Apparent K_d_ (**B**) and dissociation kinetics (**C**) determinations for Abf1BS variants. The gel images correspond to electrophoresis in a non-denaturing polyacrylamide gel; each one is representative of three independent assays. The probe used in each reaction and Abf1 concentrations are depicted at the top of gel images; migrations of free DNA probe (DNA), nucleosome probe (Nuc), DNA probe bound by Abf1 (Abf1-DNA) and nucleosome probe bound by Abf1 (Abf1-Nuc) are indicated at the right. In addition, for the dissociation kinetics analysis, the different time points and use of an unlabeled double-stranded oligonucleotide harboring an Abf1BS for Abf1 removal (chaser) are depicted at the top of the gel image. The graphs at the right of each gel image correspond to densitometric quantification of binding percentages used to calculate K_d_ (**B**) and K_off_ (**C**) values, which are displayed in Table 2

We analyzed binding strength of Reb1 and Abf1 for these variants in terms of overall affinity (apparent K_d_) and dissociation kinetics (K_off_). These analyses were performed using reconstituted mononucleosomes as probes, in order to use the same conditions subsequently used to test their influence on ISW1a’s nucleosome sliding activity. The probes harbor the 147 bp 601 nucleosome positioning sequence and 80 bp of extranucleosomal DNA (from here referred to as linker DNA) downstream the 601 sequence. In each probe, the binding site variant is located in the linker region, 10 bp downstream the 601 sequence (Figs. 1 A and 2 A). For both GRFs, the mobility shift generated on by these factors on the probes at the form of naked DNA was very similar to that generated on nucleosome probes, being only distinguishable in the case of Abf1 (Figs. S1A and S2A).

**Table 1:** K_d_, K_off_ and half-life values for Reb1 binding site variants

Probe	K_d_ (nM)	K_off_ (min.^−1^)	Half-life (min.)
**T**TTACCCG**AG** (T-AG)	5.94 ± 0.58	ND	< 0.5
**G**TTACCCG**GG** (G-GG)	1.06 ± 0.29	0.0098 ± 0.0005	76.5 ± 4.2
**G**TTACCCG**GC** (G-GC)	1.07 ± 0.25	0.0039 ± 0.0010	229.7 ± 37.4
**G**TTACCCG**TC** (G-TC)	1.11 ± 0.28	0.0408 ± 0.0038	16.2 ± 1.2

Highlighted letters in binding site sequences correspond to those that define probe name. Values indicate the average and 1 S.D. of three independent assays.

**Table 2:** K_d_, K_off_ and half-life values for Abf1 binding site variants

Probe	K_d_ (nM)	K_off_ (min.^−1^)	Half-life (min.)
**T**ATCGTATTGCATGATA**T** (T-T)	2.52 ± 0.31	0.8030 ± 0,0556	0.3 ± 0.01
**A**ATCGTATTGCATGATA**T** (A-T)	2.39 ± 0.24	0.8283 ± 0,0209	0.2 ± 0.02
**T**ATCGTATTGCATGATA**G** (T-G)	2.49 ± 0.05	ND	< 0.2
TATCGTAT**AAAG**TGATAG (A_3_G)	2.22 ± 0.14	0.0205 ± 0,0021	33.7 ± 3.70

Highlighted letters in binding site sequences correspond to those that define probe name. Values indicate the average and 1 S.D. of three independent assays.

In the case of Reb1, all binding site variants tested comply with the consensus sequence NTTACCCKN found in the JASPAR database (central position underlined; [18]), differing at positions −4, +4 and +5; the variants tested are named based in these positions (Fig. 1A). Position +5 stands as a flanking position, as it is commonly not included within the consensus Reb1 target sequence in mainstream TFBS databases, such as JASPAR, CIS-BP, YeTFaSCo and MotifMap [18–21]. Nevertheless, this position appears as part of the consensus binding sequence in a number of genome-wide analyses [11, 22–24]. Our K_d_ determinations show that the presence of T and A at positions −4 and +4, respectively (T-AG probe), results in a K_d_ nearly 5 times higher than that obtained by the presence of G in these positions (G-GG probe, Figs. 1B and S1B; Table 1). Regarding position +5, G and C (G-GG and G-GC probes) gave the same K_d_ value, at least in the sequence context tested in our analysis (Figs. 1B and S1B; Table 1). Consistent with our K_d_ determinations, our dissociation kinetics analyses showed a very short residence time of Reb1 binding for the T-AG variant (Fig. 1C), with a complex half-life below 30 s (Fig. S1D; Table 1), while the other three variants displayed markedly longer dwell times (Fig. 1C; Table 1). Differences in dwell time were also observed within these three variants. In this regard, the presence of C, A or T at position +5 has been associated with strong Reb1 binding sites in a previous genome-wide study performed by Rossi and co-workers [11]. Consistently, we found that C at position +5 (G-GC variant) confers a binding strength higher than that conferred by G at this position (G-GG variant), since the former variant displays a lower K_off_ value and a longer half-life than the latter one (Figs. 1C and S1C; Table 1). Interestingly, this long dwell time given by the presence of C at position +5 is significantly reduced when switching from G to T at position +4 (Figs. 1C and S1C; Table 1; compare G-GC to G-TC probe).

Regarding Abf1 binding site variants tested, they comply with the consensus sequence NCGTNNN**N**NRNKMBNN found in the JASPAR database (central position in bold; [18]). Three of them are derivatives of the sequence TATCGTATT**G**CATGAT (the underlined section of these sequences corresponds to their equivalent stretch), which was deducted as a consensus sequence from a SELEX approach [25] and used in our previous study [17]. These three variants differ in positions −9 and +8 (T-T, A-T and T-G variants; Fig. 2A). Position −9 is present in the consensus sequence of the SELEX study, but commonly absent in other studies and databases. On the other hand, the presence of G or C in position +8 has been associated to a biased enrichment resulting from a preferential cross-linking reaction generated by these nucleotides in that position, rather than resulting from contribution to binding strength [11]. An additional variant harbors the sequence AAAG from positions −1 to +2 (A_3_G variant, Fig. 2A). Although the region is not part of the most conserved positions of the consensus Abf1 binding site (Fig. 2A), we aimed to test this sequence variant as it is present in a higher frequency in databases and recent studies, relative to the SELEX consensus sequence [15, 24]. Under our assay conditions, all variants displayed similar K_d_ values, ranging from 2 to 2.5 nM (Figs. 2B and S2B; Table 2). The A_3_G variant displayed a slightly lower K_d_ value. Although this difference was statistically non-significant, this variant consistently displayed the longest dwell time among the variants tested in our dissociation kinetics analyses (Figs. 2C and S2C; Table 2). Indeed, for the other variants we needed to test much shorter time points, in order to determine K_off_ and half-life values (Fig. 2C). Differences in complex half-life were also observed between these variants. In this regard, the T-G variant displayed a dwell time shorter than that displayed by the T-T variant (Fig. 2C; Table 2), indicating that the presence of G at position +8 has a negative effect on binding strength. This result is consistent with previous studies showing that its enrichment in assays based on immunoprecipitation of cross-linked material does not reflect contribution to binding strength [11].

### Binding site variants featuring long dwell times endow Reb1 and Abf1 with the ability to hinder ISW1a’s sliding activity

We next tested if the binding site variants displaying low K_d_ values and/or long dwell times confer the ability of hindering ISW1a’s sliding activity to Reb1 and Abf1. To do this, we performed nucleosome sliding assays employing the same probes used in affinity and dissociation kinetics analyses. Our nucleosome probes were reconstituted by octamer transfer, implicating that they contain non-labeled donor oligonucleosomes in a large excess relative to the nucleosome probe. These oligonucleosomes serve as stringency for GRF binding and define the incubation periods used in our remodeling assays (see Methods for details).

**Fig. 3:**
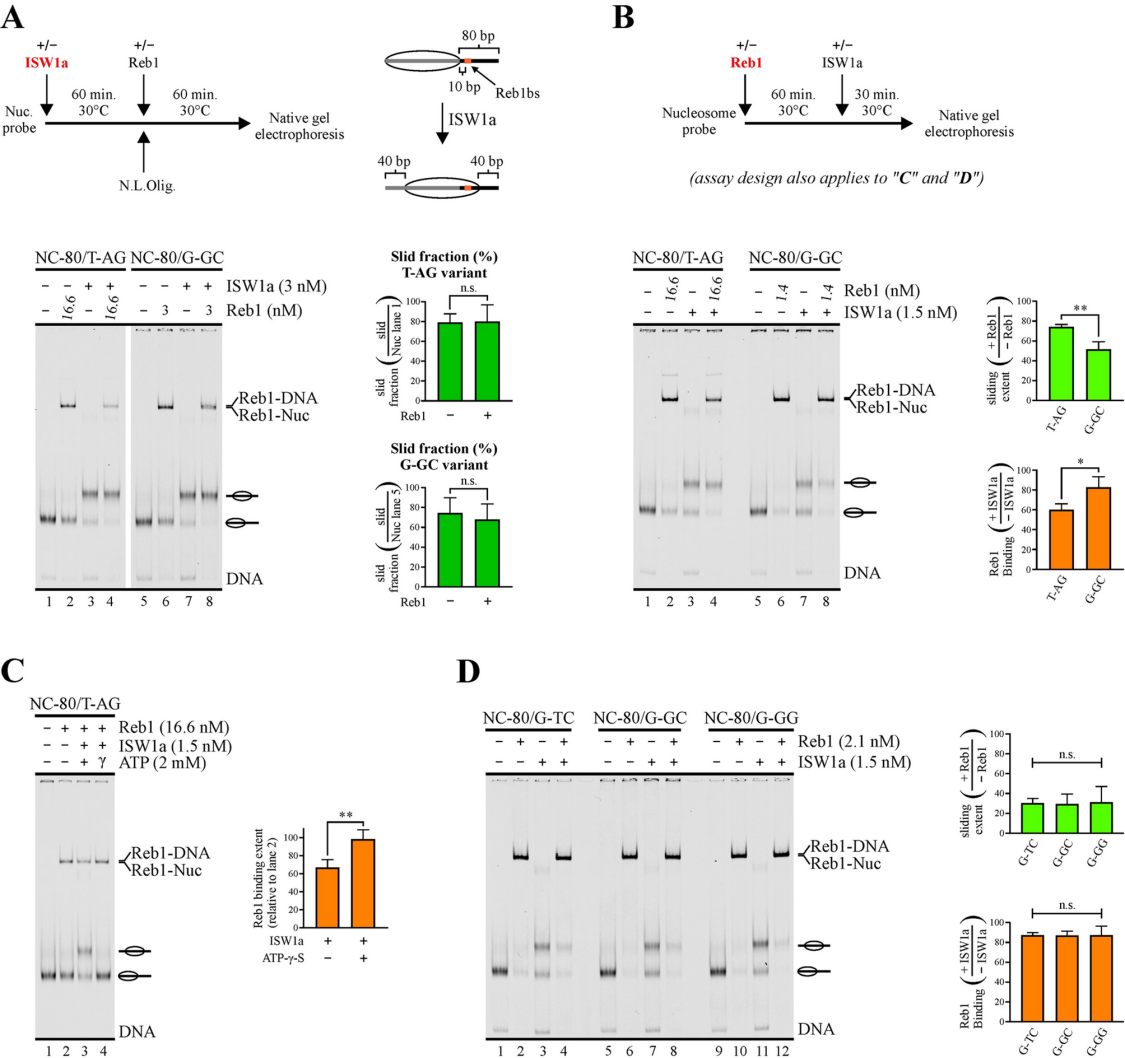
Binding site variants featuring long dwell times endow Reb1 with the ability to hinder ISW1a’s sliding activity. (**A-D**) Nucleosome sliding assays performed for the set of probes harboring Reb1BS variants (see Fig. 1A and its legend for a detailed description of the variants tested and probes design). The gel images correspond to electrophoresis in a non-denaturing polyacrylamide gel and each one is representative of three (**A, B, D**) or four (**C**) independent assays. The probe used in each reaction, presence of Reb1, ISW1a, ATP and ATP-γ-S, as well as Reb1 concentrations, are depicted at the top of gel images; migrations of free DNA probe (DNA), DNA probe bound by Reb1 (Reb1-DNA) and nucleosome probe bound by Reb1 (Reb1-Nuc) are indicated at the right, where slid and non-slid nucleosome probe populations are represented schematically. The graphs at the right of gel images correspond to determinations of sliding extent and percentage of Reb1 binding; all values used for these determinations were obtained from densitometric analyses of the corresponding gel scans. Bars in the graphs display the average of three or four independent assays for each condition analyzed. Error bars represent one standard deviation. Asymmetric connectors between bars correspond to two-tailed unpaired *t*-tests, while symmetric connectors correspond to ANOVA with Tukey’s multiple comparisons tests. Asterisks denote statistically significant differences (* *p* < 0.05; ** *p* < 0.01); n.s. = non-significant difference. (**A**) Analysis of Reb1 binding to T-AG and G-GC nucleosome probe variants after nucleosome sliding mediated by ISW1a. *Upper left*: outline of the steps involved in the assay; N.L.Olig. = non-labeled oligonucleosomes, used for ISW1a removal after nucleosome sliding mediated by this complex. *Upper right*: Schematic representation of the translational position of the Reb1 binding site resulting upon nucleosome sliding mediated by ISW1a. (**B**) Analysis of ISW1a’s sliding activity and Reb1 binding to T-AG and G-GC nucleosome probe variants, where ISW1a was added to the reactions after incubation with Reb1. *Upper panel*: outline of the steps involved in the assay. (**C**) Analysis of the effect of ISW1a action on Reb1 binding in the presence of ATP or ATP-γ-S. See outline in (**B**) for the steps involved in the assay. (**D**) Analysis of ISW1a’s sliding activity and Reb1 binding to Reb1BS variants displaying long dwell times (G-TC, G-GC and G-GG), where ISW1a was added to the reactions after incubation with Reb1. See outline in (**B**) for the steps involved in the assay. Direct measures of sliding extent and Reb1 binding percentage are presented in Figs. S3 and S5

It is currently conceived that low-affinity binding sites at gene regulatory regions, displaying short dwell times, are able to act as functional sites under conditions where a high local cognate transcription factor concentration results in binding saturation levels similar to that obtained on high-affinity sites under lower transcription factor concentrations [26, 27]. To determine whether this principle applies to the molecular function of hindering nucleosome sliding activity, for Reb1 we compared the variant displaying the lowest affinity (T-AG) to that harboring the strongest binding site (G-GC). The comparative analyses were carried out using for the T-AG probe a Reb1 concentration higher than that used for the G-GC probe, in order to compare these variants under the same binding saturation levels. The electrophoretic step of the analyses was performed without prior removal of Reb1 binding, in order to assess both sliding and Reb1 binding extent. Importantly, to directly quantify the extent of sliding activity from the bands corresponding to slid nucleosome using this approach, it was critical to determine in advance whether the GRF is able to bind the slid nucleosome in our probes. To do this, we performed assays were the probes were first incubated with ISW1a, adding Reb1 to the reactions afterwards (Fig. 3A). As the probes harbor 80 bp linker in only one side of the nucleosome core and ISW1a slides the core towards a central position [28], in the slid nucleosome the binding site resides inside the less accessible nucleosome core region (Fig. 3A). As expected for this design, no reduction in the intensity of the band corresponding to the slid nucleosome in the presence of Reb1 was observed, even in the case of the probe harboring the binding site variant of the highest affinity, G-GC (Fig. 3A). Consistently, a reduction in Reb1 binding extent upon ISW1a-mediated nucleosome remodeling was observed, with the remaining binding signal originated essentially from binding to non-slid probe and naked DNA (Figs. 3A and S3A). Thus, for this probe design, Reb1 is not able to bind to its cognate sequence in the slid nucleosome. We then tested the ability of Reb1 to hinder ISW1a’s sliding activity, comparing the same low- and high-affinity probes, by performing assays where the probes were first incubated with Reb1, adding ISW1a afterwards. As observed in Fig. 3B, only a slight Reb1-mediated hindering of sliding activity was observed for the low affinity variant, T-AG. In contrast, a significantly stronger hindering of sliding activity was observed for the probe harboring the high affinity binding site variant, G-GC (Figs. 3B and S3B). The same result was obtained by including a step of Reb1 binding removal before the electrophoretic analysis (Fig. S4B). Importantly, the G-GC variant displayed this stronger hindering even though the Reb1 concentration used for it was more than 10 times lower than that used for the T-AG probe (Fig. 3B). In terms of binding patterns, the low-affinity (T-AG) probe displays a significant reduction in the extent of Reb1 binding, while variations in Reb1 binding levels were non-significant for the high-affinity (G-GC) probe (Figs. 3B and S3B).

The result of our analysis of Reb1 binding after ISW1a-mediated nucleosome sliding (Fig. 3A) indicates that the reduction of Reb1 binding observed in Fig. 3B for the T-AG probe arises from generation of the slid nucleosome, where the Reb1 binding site is less accessible. Continuous competition between this GRF and ISW1a for interaction with linker DNA would also result in a similar effect. To test this possibility, we performed an analysis for the low affinity (T-AG) probe equivalent to that shown in Fig. 3B, but including a reaction in the presence ATP-γ-S, a non- hydrolysable analog of ATP. In the presence of this analog we observed no reduction in Reb1 binding owing to the presence of ISW1a (Fig. 3C), supporting the decreased accessibility of the binding site in the slid nucleosome as the mechanism underlying the reduction in Reb1 binding upon ISW1a-mediated nucleosome remodeling.

We next compared the effect on ISW1a’s sliding activity exerted by the Reb1BS variants displaying dwell times in the order of minutes (G-TC, G-GC and G-GG variants, Table 1). These variants displayed no differences in their ability to hinder ISW1a’s sliding activity. Consistently, Reb1 binding was only slightly reduced and in a similar extent for all these variants (Fig. 3D; Fig. S5).This analysis was performed using a higher binding saturation level (near 90%). Under this condition, further hindering of nucleosome sliding is observed for the G-GC variant, as compared to that found using lower binding saturation (Fig. 3B). Importantly, this stronger hindering was obtained using a Reb1 concentration still substantially (8 times) lower than that used for the low affinity Reb1BS (T-AG) variant (compare Fig. 3B and D).

**Fig. 4:**
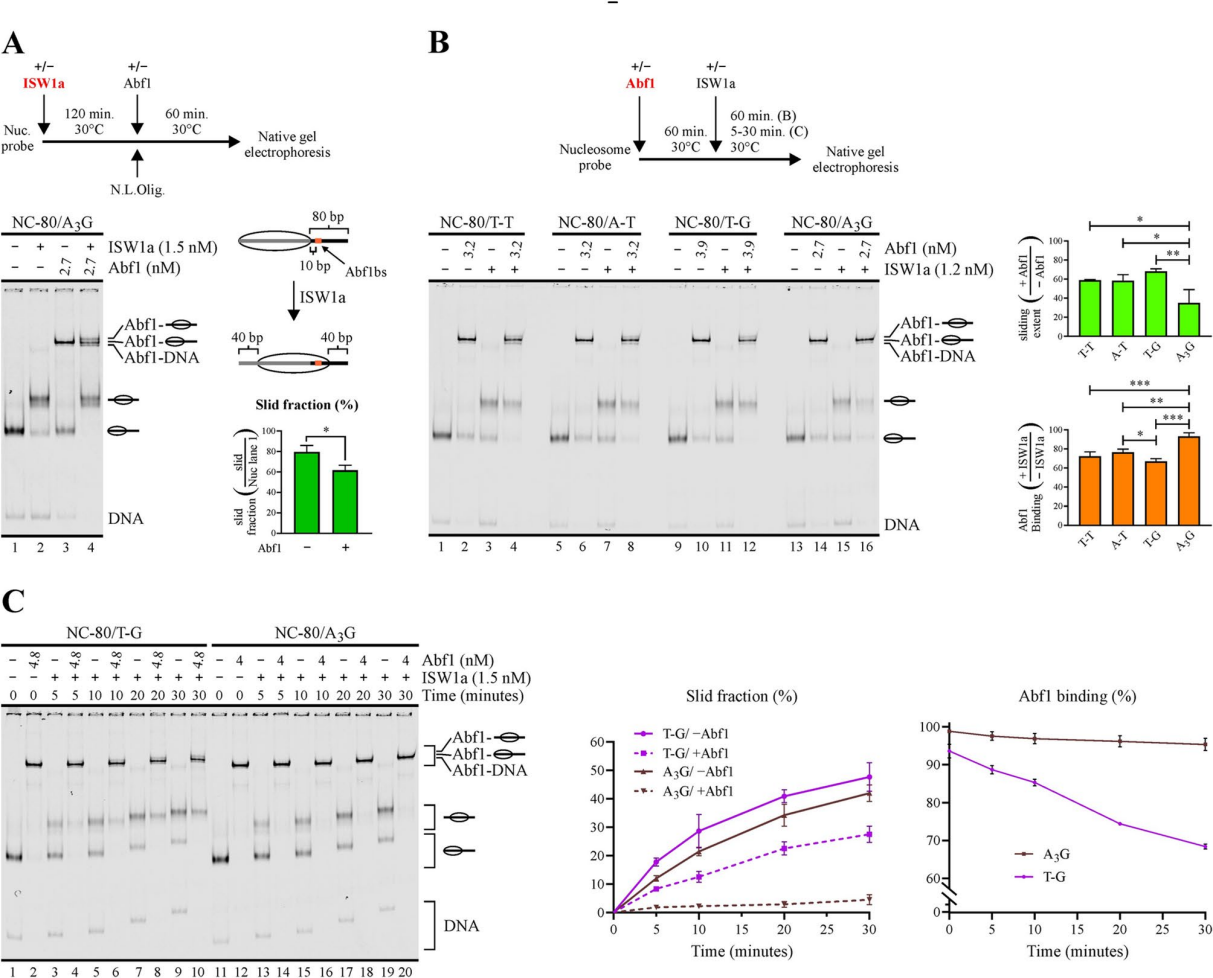
The binding site variant harboring the longest dwell time endow Abf1 with the strongest ability to hinder ISW1a’s sliding activity. (**A-C**) Nucleosome sliding assays performed for the set of probes harboring Abf1BS variants (see Fig. 2A and its legend for a detailed description of the variants tested and probes design). The gel images correspond to electrophoresis in a non-denaturing polyacrylamide gel and each one is representative of three independent assays. The probe used in each reaction, presence of Abf1 and ISW1a, as well as Abf1 concentrations, are depicted at the top of gel images; migrations of free DNA probe (DNA), DNA probe bound by Abf1 (Abf1-DNA) and nucleosome probe bound by Abf1 are indicated at the right, where slid and non-slid nucleosome probe populations are represented schematically. The graphs at the right of gel images correspond to direct measures of sliding activity (slid fraction, **A** and **C**), determinations of sliding extent in the presence of Abf1 relative to its absence or Abf1 binding upon ISW1a-mediated nucleosome sliding relative to its absence. All values used for these determinations were obtained from densitometric analyses of the corresponding gel scans. Bars in the graphs display the average of three independent assays for each condition analyzed. Error bars represent one standard deviation. Connectors between bars correspond to ANOVA with Tukey’s multiple comparisons tests, with asterisks denoting statistically significant differences (* *p* < 0.05; ** *p* < 0.01; *** *p* < 0.001). Additional analyses related to this figure are presented in Figs. S6, S7 and S8. (**A**) Analysis of Abf1 binding to the A_3_G nucleosome probe variant after nucleosome sliding mediated by ISW1a. *Upper panel*: outline of the steps involved in the assay; N.L.Olig. = non-labeled oligonucleosomes, used for ISW1a removal after nucleosome sliding mediated by this complex. *Right panel*: Schematic representation of the translational position of the Abf1 binding site resulting upon nucleosome sliding mediated by ISW1a. (**B**) Analysis of ISW1a’s sliding activity and Abf1 binding to Abf1BS variants, where ISW1a was added to the reactions after incubation with Abf1 and nucleosome sliding incubations were conducted for 60 min. (**C**) Time-course analysis, ranging from 5 to 30 min of the nucleosome remodeling incubation

We then proceeded to compare the Abf1BS variants in terms of the ability of Abf1 to hinder ISW1a’s sliding activity. Unlike the case of Reb1, where one of the binding site variants displayed a markedly higher K_d_ value, the binding affinity of Abf1 for the variants tested appeared to be very similar (Fig. 2A, Table 2). However, as detailed above, marked differences were obtained in terms of dwell time, with the A_3_G variant displaying the longest time, with a half-life in the order of minutes, followed by T-T and the A-T variants displaying half-life values of only few seconds (Fig. 2B, Table 2). As in the case of Reb1, we first tested whether Abf1 binds to the slid nucleosome probe, by first incubating with ISW1a and adding Abf1 to the reactions afterwards. This analysis was performed for the variant displaying the longest dwell time (A_3_G; Fig. 4A). Interestingly, Abf1 displayed interaction with the slid nucleosome, reflected by the appearance of a faint band of slightly lower migration than that given by Abf1 interaction with the non-slid nucleosome probe (Fig. 4A). The binding extent of Abf1 to the slid nucleosome was markedly weaker than that displayed by this GRF to the same probe at the form of naked DNA or non-slid nucleosome (Fig. 4A), being therefore discarded in calculations of sliding extent of remodeling assays. Nevertheless, the appearance of this band served as an additional indicator of the differences displayed by the Abf1BS variants in terms of their ability to hinder ISW1a’s sliding activity (see below). The result of the nucleosome remodeling assay showed that Abf1 hinders ISW1a’s sliding activity in all variants, with the binding site variant of the longest dwell time (A_3_G) displaying the most pronounced hindering and the lowest reduction of Abf1 binding, while the variant of the shortest dwell time (T-G) displayed the weakest hindering of ISW1a’s activity, accompanied by the most pronounced reduction in Abf1 binding (Figs. 4B, S6 and S7). Importantly, the faint band reflecting Abf1 binding to the slid nucleosome appeared for all variants, except in the case of the A_3_G variant, further confirming the higher ability of Abf1 to hinder ISW1a’s sliding activity in the case of this variant (Fig. 4B and C and S6B). By performing a 30 min nucleosome remodeling incubation, as was the case of the analyses performed for Reb1, differences between the variants harboring intermediate half-life values (T-T and A-T) and the A_3_G variant were statistically non-significant (Fig. S6B). In light of this result, we reasoned that a longer incubation after adding the CRC in the remodeling assay could result in more marked differences between these variants, considering that more dissociation events would occur during this longer incubation, implying more chances for the CRC to exert its activity. Thus, we performed an additional analysis, extending the incubation after addition of ISW1a from 30 to 60 min. Under this setting, a more marked difference between the longest dwell time variant (A_3_G) and the other variants was found, being statistically significant relative to all the other variants (Figs. 4B and S7). The results obtained by extending the time of the remodeling incubation additionally suggested that in the shorter remodeling incubation period (30 min) a steady state given by a defined extent of Reb1 binding during continuous action of ISW1a was not present. To confirm this, we performed a time-course analysis from 5 to 30 min, comparing the variants displaying the shortest and longest dwell time (T-G versus A_3_G, respectively) and using Abf1 concentrations to reach over 90% binding saturation. The result of this analysis shows a continuous increment of the slid nucleosome in the absence of Abf1 during the whole incubation period. Similar to the previous analyses, in each time point the extent of sliding hindering was markedly lower for the shortest dwell time variant (T-G) than for the variant displaying the longest dwell time (A_3_G). Consistent with the results of the previous remodeling assays (30 and 60 min), in the case of the T-G variant Abf1 displayed a progressively lower sliding hindering and binding extent (Figs. 4C and S8). In addition, the results of this kinetic analysis demonstrate that the extent of sliding hindering and GRF binding in the remodeling assays of our current study do not reflect the reaching a steady state.

### The sequence pattern of variants characterized as strong sites is enriched at loci where Reb1 and Abf1 act as strong nucleosome displacement factors

We have previously shown that the binding strength of the GRF Rap1 is inversely correlated with nucleosome occupancy and histone deposition genome-wide. The findings of our current study suggested that Reb1 and Abf1 would display a similar pattern [17]. To confirm this, we analyzed the correlation between binding strength and both nucleosome occupancy and histone deposition for Reb1 and Abf1. To do this, we compared genome-wide datasets of in vivo (ChIP-exo) and in vitro (PB-exo) binding profiles of Reb1 and Abf1 obtained by Rossi et al. [11]. to genome-wide patterns of nucleosome occupancy and histone deposition generated by Kassem et al. [29]. PB-exo consists in transcription factor immunoprecipitation in the presence of only purified genomic DNA, ensuring that binding patterns are not influenced by the chromatin context [11]. Two clusters were extracted from Reb1 binding peaks as well as for Abf1 peaks, corresponding to 20% of highest and 20% of lowest occupancy (termed Top and Bottom, respectively; Fig. S9). These two clusters displayed statistically significant differences in terms of both nucleosome occupancy and histone deposition, for Reb1 and Abf1 (Fig. S9). In addition, these two parameters were inversely correlated with occupancy of these GRFs. Similar results were obtained from both ChIP-exo and PB-exo data (Fig. S9).

**Fig. 5:**
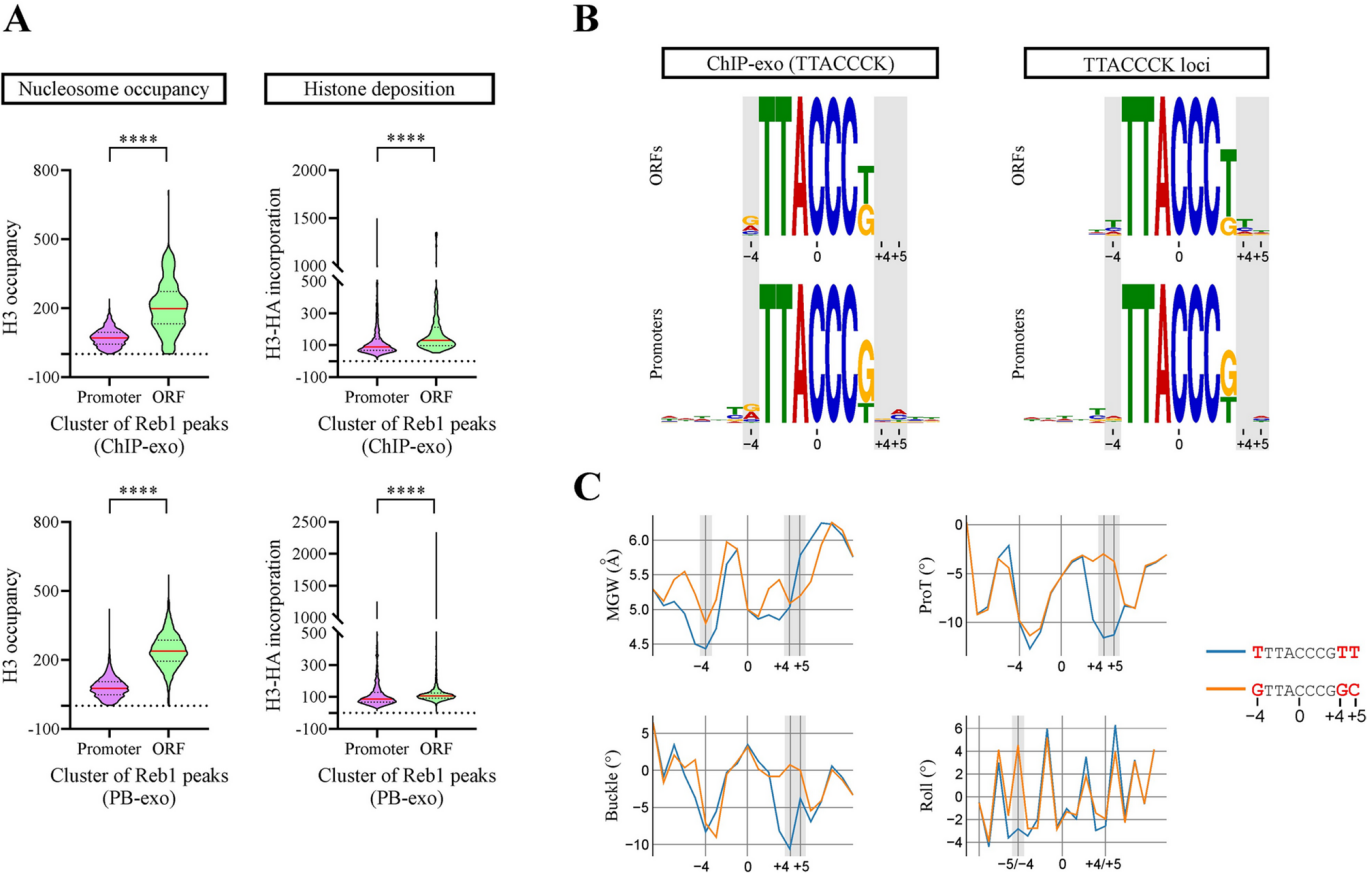
Differential sequence frequency profiles and DNA shape features are reflected by Reb1 binding sites located at gene bodies relative to promoter regions. (**A**) Violin plots comparing the distribution of nucleosome occupancy (left panel) and histone deposition (right panel) levels for Reb1 binding sites located at gene bodies (ORFs) and promoter regions, according to in vitro (PB-exo) or in vivo determinations performed by Rossi and co-workers [11]. Nucleosome occupancy (histone H3) and histone deposition levels (H3-HA incorporation) were determined from genome-wide ChIP-seq data obtained by Kassem and co-workers [29]. Asterisks denote statistically significant differences (**** *p* < 0.0001), as deducted from the Mann-Whitney *U* test. (**B**) Consensus sequence exhibited by the same clusters of Reb1 binding site. The clusters were generated using the aforementioned ChIP-exo data, filtering by sites complying with the Reb1BS core sequence (TTACCCK) or, alternatively, by collecting the loci from ORFs or gene promoters harboring this Reb1BS core sequence. The logos of consensus binding sites were generated using the MEME suite [30]. Position probability matrices are reported in Tables S3 and S4. (**C**) DNA shape features predicted for the major transition of Reb1BS sequence pattern from gene bodies to promoter regions, using the Deep DNAshape webserver [32, 33]. The graphs correspond to the most significant differences found between the sequence patterns analyzed. ProT = propeller twist

**Fig. 6:**
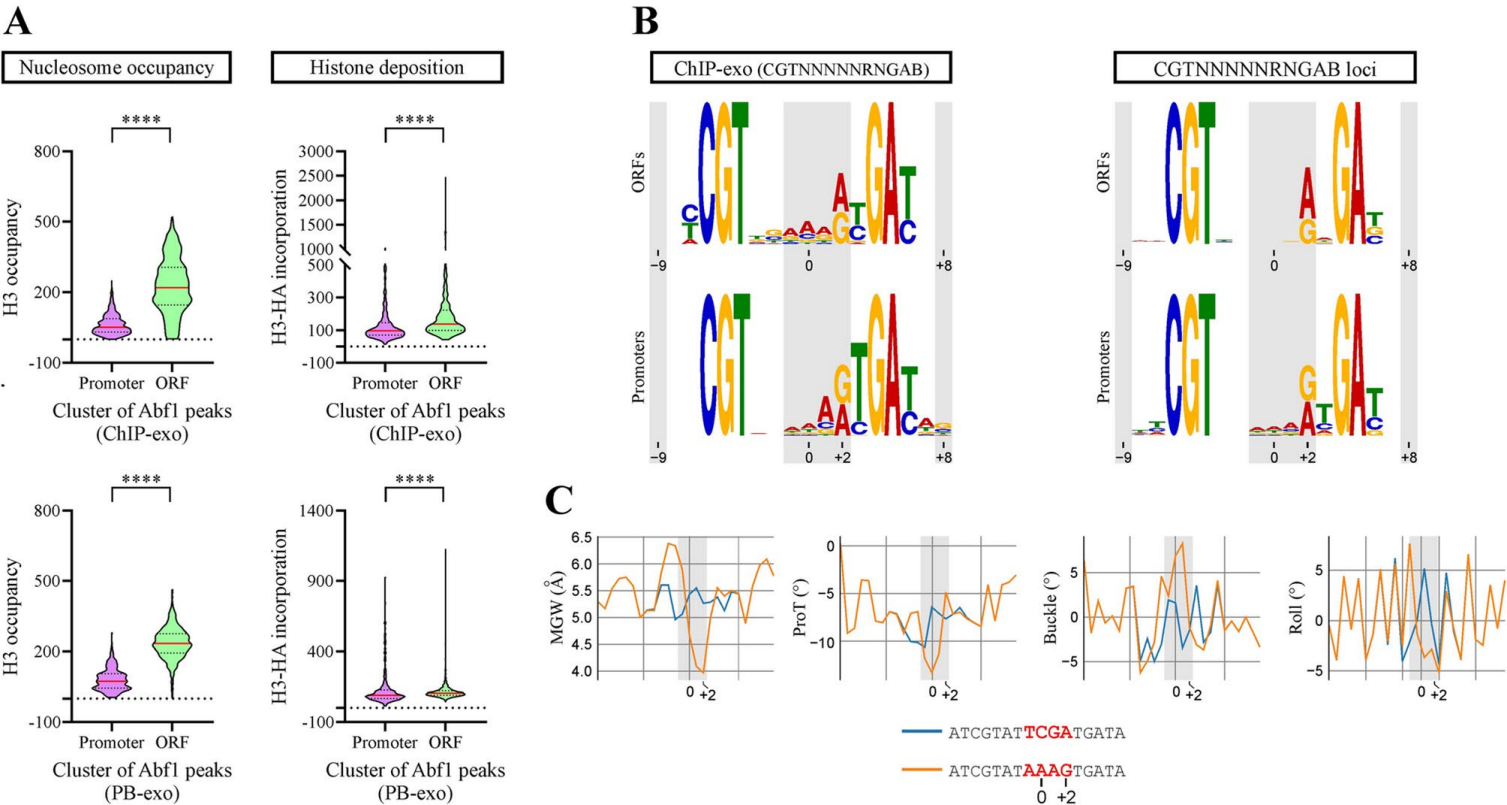
Differential sequence frequency profiles and DNA shape features are reflected by Abf1 binding sites located at gene bodies relative to promoter regions. (**A**) Violin plots comparing the distribution of nucleosome occupancy (left panel) and histone deposition (right panel) levels for Abf1 binding sites located at gene bodies (ORFs) and promoter regions, according to in vitro (PB-exo) or in vivo determinations performed by Rossi and co-workers [11]. Nucleosome occupancy (histone H3) and histone deposition levels (H3-HA incorporation) were determined from genome-wide ChIP-seq data obtained by Kassem and co-workers [29]. Asterisks denote statistically significant differences (**** *p* < 0.0001), as deducted from the Mann-Whitney *U* test. (**B**) Consensus sequence exhibited by the same clusters of Abf1 binding site. The clusters were generated using the aforementioned ChIP-exo data, filtering by sites complying with the Abf1BS consensus sequence (CGTNNNNNRNGAB) or, alternatively, by collecting the loci from ORFs or gene promoters harboring this Abf1BS sequence. The logos of consensus binding sites were generated using the MEME suite [30]. Position probability matrices are reported in Tables S5 and S6. (**C**) DNA shape features predicted for the major transition of Abf1BS sequence pattern from gene bodies to promoter regions, using the Deep DNAshape webserver [32, 33]. The graphs correspond to the most significant differences found between the sequence patterns analyzed. ProT = propeller twist

As mentioned in the Introduction, recent findings point to a linkage between defined GRFs’ binding site variants and their preferential occupancy at gene promoters, relative to gene bodies, while other findings show that sequence variations in flanking positions of TFBSs could deeply affect TFs’ binding strength [11–13]. Considering this, we aimed to determine nucleosome occupancy and histone deposition patterns for Reb1 and Abf1, now comparing their binding sites located at gene bodies (ORFs) to their sites located at gene promoters. These clusters were compared using the same data and methodology described in the previous paragraph. As expected, lower histone occupancy levels are observed for the binding sites located at gene promoters (Figs. 5A and 6A). Regarding histone deposition, it is intimately linked to histone exchange, which is in general higher at gene promoters than at gene bodies [29]. Interestingly, our analysis show that binding sites of both Reb1 and Abf1 located at gene bodies display slightly higher levels of histone deposition than those observed at gene promoters (Figs. 5A and 6A). To ascertain whether these differential nucleosome occupancy and histone deposition levels were correlated to defined sequence patterns of Reb1 and Abf1 binding sites, we determined the consensus binding sites exhibited by the gene body and gene promoter clusters, using the MEME suite [30]. For Reb1, the analysis based in ChIP-exo data shows that the high frequency of G at position +4 and C at position +5 for the consensus sequence obtained from the whole genome is contributed by gene promoters (Fig. S10A). Similarly, enrichment of A at position +1 and G at +2 of Abf1BS is contributed by gene promoters (Fig. S10B). Moreover, the same result is obtained when filtering these two clusters by those peaks that comply with the core sequence of Reb1 (TTACCCK) or Abf1 (CGTNNNNNRNGAB) binding sites (Figs. 5B and 6B; Tables S3 and S5). A similar pattern was observed by performing the analysis using the PB-exo data (Fig. S11). Finally, we analyzed differences in the consensus binding site displayed by promoters relative to gene bodies by simply collecting all loci that comply with the core sequence of Reb1BS (TTACCCK) and Abf1 (CGTNNNNNRNGAB) and separated them in promoter and gene body clusters. Then, we determined the consensus binding site for each cluster and analyzed changes in sequence frequency in less conserved positions, obtaining results consistent with those obtained from analysis of ChIP-exo and PB-exo data. For Reb1, the results show that the frequency of T at positions −4 and +4 of gene promoter sites is markedly lower than that found in ORF sites, while the opposite occurs with the frequency of G at the same positions. In addition, at position +5 the highest increment in frequency from gene bodies to promoters corresponded to the appearance of C (Fig. 5B; Table S4). For Abf1, gene promoters display a higher frequency of A at position +1 and an A to G transition from ORFs to promoters at position +2 (Fig. 6B; Table S6).

There is growing evidence showing that sequences flanking TFBSs affect their shape and conformational flexibility, which in turn can influence on transcription factor affinity for its target sequence [13, 31]. Considering this, we aimed to analyze the influence of flanking and low conservation positions on DNA shape parameters. To do this, we used the Deep DNAshape webserver [32, 33], focusing on shape differences for the main sequence transitions found in our genome-wide analyses. The results show marked differences in several shape features for both Reb1 and Abf1. For Reb1, the features displaying the most pronounced differences were minor groove width, propeller twist, buckle and roll, given mainly by positions −4 and/or +4/+5 (Fig. 5C). The same shape features displayed the most pronounced differences in the case of the Abf1, given by transition from TCGA to AAAG at the −1 to +2 region (Fig. 6C).

## Discussion

In this work we have found that sequence changes at positions of low conservation of Reb1 and Abf1 binding sites could deeply affect the binding strength of these GRFs, affecting dwell time and the ability to hinder the sliding activity of the ISW1a complex. Importantly, strong binding sites displayed a significantly higher ability to hinder ISW1a’s sliding activity, even under conditions where a markedly higher GRF concentration for the weak binding site was used to compare weak to strong binding sites under equal binding saturation levels. Moreover, genome-wide analyses showed that the sequence variants that confer the ability to hinder sliding activity to Reb1 and Abf1 are enriched at promoter regions relative to gene bodies.

Regarding the analyses of Reb1BS variants, position +5 displayed a strong effect on dwell time. As mentioned above, this position is commonly not included within the consensus Reb1 target sequence in mainstream TFBS databases. Besides this, position +4 displayed an even more pronounced effect on this parameter, with the presence of T resulting in a significant reduction in dwell time. Consistently, we found that the frequency of T at this position is markedly higher at ORFs than at gene promoters. It remains to analyze the specific effect of position −4 on binding parameters and effect on sliding activity, having no variations at other positions. Regarding Abf1BS variants, we observed that the presence of G at position +8 has a negative effect on binding strength, with a reduction in residence time. On the other hand, the sequence AAAG at the central portion of the binding site overcomes this effect and results in a markedly higher dwell time. Consistently, a previous study performed by Jonge et al., addressing in vivo dwell time for Abf1, found the same sequence pattern enrichment at loci displaying the longest residence time [15]. Within this context, it is worth noting that the Reb1BS variants harboring dwell time in the order of minutes display a similar ability to hinder ISW1a’s sliding activity. On the other hand, the ability to hinder sliding activity displayed by the Abf1BS variant harboring a dwell time in the order of minutes was significantly higher than that of the Abf1BS variants that harbor dwell times in the order of seconds. These results suggest that residence times in the order of minutes would be sufficient for the task of hindering sliding activity, at least in the case of ISW1a. In addition, in the case of Abf1, the half-life value found in our in vitro dissociation kinetics analyses for the variant displaying the longest dwell time falls close to the residence time found for this GRF in the study performed by de Jonge et al. [15].

Interestingly, our genome-wide analyses show that binding sites of both Reb1 and Abf1 located at gene bodies display slightly higher levels of histone deposition than those observed at gene promoters. It is known that replication-independent histone deposition, which is connected to histone exchange, is in general higher at gene promoters than at gene bodies [29]. In this regard, we also found that defined sequence patterns are enriched at gene promoters and that these patterns correlate with long dwell time binding. Therefore, our results regarding histone deposition suggest that Reb1 and Abf1 display the property of hindering histone deposition in the vicinity of their target regions, even at gene promoters, if the bound region harbors a strong binding site variant.

The different techniques used by strategies developed to determine consensus binding sites for transcription factors (protein binding microarrays; HT-SELEX; ChIP-seq and others) give the possibility to determine the affinity for binding site variants from the position frequency matrices [34]. The results of our genome-wide analyses suggest that strategies based on ChIP-seq or similar techniques could be strengthened by adding determinations of consensus binding sites from gene promoters and, separately, from gene bodies, besides the generation of consensus binding sites gathered from the whole genome. This data processing would benefit different types of studies that make use of the information deposited in TFBS databases. Besides this, strategies based on ChIP-seq developed to assess in vivo dwell time [15] stand as appropriate alternative approaches to determine high affinity binding site variants. In this context, studies that use TFBSs information would also benefit from progresses in methodologies designed to predict affinities to binding site variants, particularly in the case of flanking sequences. Methods that consider DNA shape features to predict TFBSs [35, 36] and methods designed to predict protein and protein-DNA complex structures [37, 38] have recently been developed. In addition, recent studies have addressed binding preferences from protein structure and DNA shape features [31, 39]. The combined use of protein structure prediction, DNA shape/flexibility and relative affinities for binding site variants will significantly enrich TFBS information and contribute to the studies that make use of this type of resources.

Diverse studies have shown that, even on low-affinity binding sites, TFs can perform their molecular functions, provided that a high local concentration of the TF is present [26, 27]. In this regard, our results suggest that this principle would not apply for the molecular function of hindering nucleosome sliding activity. As mentioned above, we observed that, under equal binding saturation levels for weak and strong binding sites, obtained by using markedly higher TF concentration for the low-affinity binding site, the latter still display a significantly lower ability to hinder nucleosome sliding activity. This fact indicates that, even under elevated binding saturation levels, the high frequency of dissociation events allowed by weak binding sites would maintain abundant windows of opportunity for a CRC-mediated nucleosome sliding towards the NFR. Therefore, it will be of relevance to define those molecular functions that require long dwell times to be accomplished and those that do not require this property, complemented by determining the TFs responsible for these functions. Other molecular functions that might rely on long dwell times would be stimulation of nucleosome eviction [6] and hindering of histone deposition [17, 29]. In this context, we have recently shown that poly(dA:dT) tracts have the property of hindering ISW1a’s sliding activity [40]. Considering this, it will be of relevance to determine whether defined DNA sequences, such poly(dA:dT) tracts, would grant low- to medium-affinity binding sites with the ability to hinder nucleosome sliding to levels similar to those displayed by high-affinity binding sites. Taken together, these advances will significantly contribute to our knowledge of the syntax of *cis*-regulatory elements.

## Conclusions

The frequency of nucleotides of flanking or less-conserved positions of Reb1 and Abf1 binding sites vary from gene bodies to promoter regions. Sequence variation at some of these positions strongly affect binding affinity, particularly residence time. Importantly, this is a key feature that grants the ability to hinder nucleosome sliding activity to these GRFs. Moreover, hindering of nucleosome sliding is not a molecular function where low-affinity binding sites under high transcription factor concentrations can replace the function of high-affinity binding sites. To determine those molecular functions that require long dwell times and the TFs responsible for these tasks will entail a significant contribution to the efforts being made to establish the grammar of *cis*-regulatory elements.

## Methods

### Recombinant proteins and protein complexes

His-tagged Reb1 and Abf1 were purified as recently described by us [17]. The eluted fractions were supplemented with glycerol (15% final concentration) and then dialyzed against 500 volumes of dialysis buffer [10 mM Hepes-KOH (pH 7.9), 300 mM NaCl, 1 mM DTT, 0.5 mM PMSF, 10 µM ZnCl_2_, 15% Glycerol, 10 mM Imidazole] using Slide-A-Lyzer Dialysis Cassettes (ThermoFisher). After dialysis, proteins were supplemented with pepstatin A (1 µg/mL, final concentration) and leupeptin (5 µg/mL, final concentration) and then stored at −80 °C until their use. The ISW1a complex was obtained by tandem affinity purification from an Ioc3-TAP *S. cerevisiae* strain (YFR013w, Open Biosystems), as previously described [41]. The purified complex was analyzed as previously reported by us [42]. For each complex, an aliquot of a purification was extensively concentrated (Microcon Ultracel YM-10, Amicon-Millipore), quantified by SDS–PAGE followed by Coomassie staining and then used as standard for Western blot quantification of the purified complex in that and further purifications.

### DNA probes and nucleosome reconstitution

Plasmids bearing variants of Reb1 and Abf1 binding sites were generated by inserting cassettes containing Reb1 or Abf1 binding site variants in vectors harboring Widom’s 601 nucleosome positioning sequence. The 147 bp positioning region of the 601 sequence was defined as previously described [43]. In vectors generated for all binding site variants the 601 sequence is separated from the binding site by 10 bp. These plasmids were then used as PCR templates to generate the DNA probes. These probes were labeled by including a fluorescently labeled forward primer (IRDye700, IDT) in the PCR reactions. DNA probes consisted of 227 bp, harboring the 601-nucleosome positioning sequence and Reb1 or Abf1 binding site variants. After PCR reactions, PCR products were purified from DNA polymerase, excess dNTPs and primers using an E.Z.N.A. PCR clean up kit (Omega Bio-tek), following manufacturer’s instructions. The plasmids and primer sets used are depicted in Table S1.

Nucleosome reconstitution was carried out by the octamer transfer method, as previously described by us [40]. Oligonucleosomes used as histone donors for reconstitution were obtained from chicken erythrocytes as previously described [44], using 0.6 M NaCl concentration in dialysis and chromatography buffer. All the reconstitution reactions were carried out using 0.5 pmol of probe and 1.5 µg of oligonucleosomes. Once reconstituted, the nucleosome probe (and mock-reconstituted probe) concentration is 4 fmol/µL and the non-labeled oligonucleosomes concentration is 12 ng/µL, in terms of DNA content (90 fmol/µL approx., in terms of nucleosome units).

### Binding assays

In each binding reaction, a mix containing 7.9 µL of Remodeling buffer, 0.6 µL of ddH_2_O, 2 µL of GRF (diluted in TF buffer) or TF buffer, 2 µL of CRC buffer and 2.5 µL of probe was incubated for 1 h and 30 min at 30 °C (see the composition of each solution used in the mixes in the Supplementary Information). The final concentration of the different components of this mixture was: 11.87 mM HEPES-KOH pH 7.9, 3 mM Tris-Cl (pH 7.4-8.0), 96.67 mM NaCl, 3.33 mM KCl, 5.3 mM MgCl_2_, 0.13 mM Mg(CH_3_COO)_2_, 0.05% NP-40, 10% glycerol, 1.33 µM ZnCl_2_, 0.17 mM EDTA (pH 8.0), 0.27 mM EGTA, 100 µg/mL BSA, 1.47 mM imidazole, 2 mM DTT, and 0.5 mM PMSF. The samples were then subjected to electrophoresis in non-denaturing polyacrylamide gel (200 V, 0.3x TBE, 5% acrylamide, 40:1 AA:Bis proportion) in cold room. Afterwards, the gel was scanned using Odyssey CLx Imaging System (LICORbio). Densitometric analyses were performed using Image Studio™ 6.0 Software (LICORbio). The extent of binding was calculated as the ratio of bound nucleosome band signal over the total lane signal, which corresponds to the sum of signals from bound and unbound nucleosome bands plus the free DNA band. For determination of K_d_ values, preliminary assays were performed to establish the incubation time required to reach binding equilibrium and the concentration points to be used for GRF titration. Binding incubation times tested ranged from 30 min to 3 h, defining 1 h as already sufficient to reach binding equilibrium for both Reb1 and Abf1, under our assay conditions (Fig. S12). Data fitting and statistical analysis were performed in GraphPad Prism 8. Data was fitted to a nonlinear regression curve, where the independent variable (X) was the logarithm of the concentration of GRF employed and the dependent variable (Y) was the percentage of bound GRF. Curves were constrained to a bottom value greater than 0 and a top value equal to 100.

### Dissociation kinetics

For the analysis of dissociation kinetics, the binding reaction proceeded as described for the binding analyses but scaled 6 times in volume. After binding incubation, a 15 µL aliquot was taken and 13 µL subjected to electrophoresis as described above. To the rest of the sample, 2.5 µL of non-labeled double-stranded oligonucleotide harboring the GRF’s target sequence (chaser oligos; 100x final concentration in the reaction mix, relative to the highest GRF concentration used in the assay) was added, and the incubation at 30 °C was continued for 60 min. During this incubation time, 15 µL aliquots were taken at defined time points (0.5, 2, 4 and 5 min or 5, 15, 30 and 60 min) and immediately loaded in the gel. Subsequent analyses proceeded as described above. Prior to these assays, the efficiency of chaser oligos for Reb1 and Abf1 was tested for each of the probes used by performing binding reactions in the same conditions mentioned before, but adding chaser oligos before the probe and GRF. If there was no binding of the GRF to the different probes under these conditions, then the chaser oligos were considered appropriate to perform dissociation assays (Fig. S13). To obtain the rate constant for the dissociation reaction the data were fit to the equation.

ln([complex]/[complex_o_]) = − k_off_*t*.

where the negative slope of the fit provided K_off_ values. Dwell time half-lives were determined using an exponential decay curve fitting.

### Nucleosome sliding assays

For assays where Reb1 was added after sliding by ISW1a complex (Fig. 3A), a mix containing 7.9 µL of Remodeling buffer, 0.6 µL of ATP (Roche, 11140965001), purified ISW1a complex (brought to 2 µL using CRC buffer) or CRC buffer (2 µL) and 2.5 µL of probe was incubated for 1 h at 30 °C (see the composition of each solution used in the mixes in the Supplementary Information). Then, Reb1 (diluted in TF buffer) or TF buffer (2 µL) was added, plus 60ng of long oligonucleosomes (LON), incubating for 1 h at 30 °C. After this incubation, samples were immediately subjected to gel electrophoresis (200 V, 0.3x TBE, 4% acrylamide, 40:1 AA:Bis proportion).

For assays where the GRF (Reb1 or Abf1) was added before sliding by ISW1a complex (Figs. 3B-D and 4B-C), a mix containing 7.9 µL of Remodeling buffer, 0.6 µL of ATP, 2 µL of GRF (diluted in TF buffer) or TF buffer and 2.5 µL of probe was incubated for 1 h at 30 °C. Then, purified ISW1a complex (brought to 2 µL using CRC buffer) or CRC buffer (2 µL) was added and the incubation proceeded for 30 min at 30 °C. The samples were then immediately subjected to gel electrophoresis (200 V, 0.3x TBE, 5% acrylamide, 40:1 AA:Bis proportion). Densitometric analyses were performed as described above.

The extent of nucleosome sliding was calculated for each individual probe as the ratio of the signal of slid mononucleosome band, over the signal of the corresponding mononucleosome band in the lane where neither GRF nor ISW1a complex were added. GRF binding percentage was calculated as mentioned in binding assays, considering all bands detected in the lane to define the sum of total lane signal.

### Bioinformatics analyses

Gene promoters and ORFs were obtained using a table provided by Chereji et al., 2018 [45], which contains TSS, ORF start, and ORF end coordinates for the S. cerevisiae genome. Promoters were defined as 250 bp upstream the TSS. On the other hand, gff files corresponding to Filtered_PeakPairs for ChIP-exo and PB-exo of Abf1 and Reb1, and bigwig files corresponding to H3-HA incorporation [H3-HA_TBP-AA_T30 (rep2)] and H3 occupancy [H3_TBP-AA_T30 (rep2)] were obtained from GSE93662 [11] and GSE143305 [29], respectively. For bigwig files, the genome was partitioned into 50 bp bins using bigwigAverage function from deeptools, to calculate the incorporation or occupancy in these bins.

For violin plots, files were sorted, and clusters were generated through intersections between Abf1 (or Reb1) peaks with gene promoters and ORFs using bedtools Intersect option -wo. Subsequently, these clusters were intersected with H3-HA incorporation or H3 occupancy as described above and analyzed in GraphPad Prism 8.0. Presence or absence of statistically significant differences was determined using the Mann-Whitney U test. Alternatively, Abf1 and Reb1 peaks were partitioned in quintiles based on their scores and top and bottom clusters were intersected with H3-HA incorporation or H3 occupancy as previously described.

For binding site logos of Abf1 and Reb1, peaks corresponding to gene promoters and ORFs were extended by 10 bp on either side and the corresponding FASTA sequences were extracted using bedtools getfasta with the sacCer3 genome as reference. Then, we used FIMO tool from MEME Suite [30] to keep only peaks complying with CGTNNNNNRNGAB or TTACCCK sequence for Abf1 or Reb1, respectively. Subsequently, the logo motif for each transcription factor was generated using the MEME tool from the MEME Suite [30]. Alternatively, logo motif for each transcription factor was obtained for ChIP-exo datasets without sequence filtration as described above.

Additionally, FASTA sequences of gene promoters and ORFs were extracted using the coordinates provided by Chereji et al., 2018 [45], and bedtools getfasta option -name. Then, we searched for any motif complying with CGTNNNNNRNGAB or TTACCCK sequences and motif logos were generated as detailed in the previous paragraph.

DNA shape features were determined using the Deep DNAshape webserver [32, 33], using layer 5 for groove and intra-base-pair features and layer 4 for inter-base-pair features.

## Electronic supplementary material

Below is the link to the electronic supplementary material.

**Supplementary Material 1: Additional file 1**. Detailed information regarding Methods. **Figure S1**. Analyses supplementary to Fig. 1. **Figure S2.** Analyses supplementary to Fig. 2. **Figure S3.** Analyses supplementary to Fig. 3A and B. **Figure S4.** Remodeling assays using GRF’s binding removal. **Figure S5.** Analyses supplementary to Fig. 3D. **Figure S6.** Analysis supplementary to Fig. 4 (30 min remodeling incubation). **Figure S7.** Analysis supplementary to Fig. 4B. **Figure S8.** Analysis supplementary to Fig. 4C. **Figure S9.** Binding strength of Reb1 and Abf1 inversely correlate with nucleosome occupancy and histone deposition in vivo. **Figure S10** Analysis supplementary to Figs. 5 and 6 (ChIP-exo). **Figure S11** Analysis supplementary to Figs. 5 and 6 (PB-exo). **Figure S12** Binding equilibrium for Reb1 and Abf1 is reached after 1 h incubation. **Figure S13** Verification of chasing efficiency of oligonucleotides used for Reb1 and Abf1 capture in dissociation kinetics analyses. **Table S1** Sequence information of template plasmid and primers used for generation of each probe. **Table S2.** Sequence information of oligonucleotides used in dissociation kinetics analyses. **Tables S3 to S6.** Position frequency matrices for Reb1 and Abf1 binding sites.

## Data Availability

Source data of this study are available on request from the corresponding author.
