# Supplementary material for: Variations in flanking or less conserved positions of Reb1 and Abf1 consensus binding sites lead to major changes in their ability to modulate nucleosome sliding activity
Source: Biol Res. 2025 Jul 29;58:53. doi: 10.1186/s40659-025-00627-0 (PMC12305957; doi:10.1186/s40659-025-00627-0)
Supplement: Supplementary file 1 — Supplementary Material 1: Additional file 1. Detailed information regarding Methods. Figure S1. Analyses supplementary to Fig. 1. Figure S2. Analyses supplementary to Fig. 2. Figure S3. Analyses supplementary to Fig. 3A and B. Figure S4. Remodeling assays using GRF’s binding removal. Figure S5. Analyses supplementary to Fig. 3D. Figure S6. Analysis supplementary to Fig. 4 (30 min remodeling incubation). Figure S7. Analysis supplementary to Fig. 4B. Figure S8. Analysis supplementary to Fig. 4C. Figure S9. Binding strength of Reb1 and Abf1 inversely correlate with nucleosome occupancy and histone deposition in vivo. Figure S10 Analysis supplementary to Figs. 5 and 6 (ChIP-exo). Figure S11 Analysis supplementary to Figs. 5 and 6 (PB-exo). Figure S12 Binding equilibrium for Reb1 and Abf1 is reached after 1 h incubation. Figure S13 Verification of chasing efficiency of oligonucleotides used for Reb1 and Abf1 capture in dissociation kinetics analyses. Table S1 Sequence information of template plasmid and primers used for generation of each probe. Table S2. Sequence information of oligonucleotides used in dissociation kinetics analyses. Tables S3 to S6. Position frequency matrices for Reb1 and Abf1 binding sites. [file 40659_2025_627_MOESM1_ESM.pdf]

Supplementary Information

Methods

Composition of buffers mixed in binding and nucleosome sliding assays

The final concentration of each component in a reaction mix is given in the Methods section of the main text.

Remodeling buffer (7.9 µL): 20 mM HEPES-KOH (pH 7.9), 6.33 mM KCl, 0.05 % NP-40, 9.49 % Glycerol, 10.06 mM $MgCl_2$, 132.91 µg/mL BSA, 1.84 mM DTT, 0.54 mM PMSF.

His-protein or TF buffer (2µL): 10 mM Hepes-KOH (pH 7,9), 300 mM NaCl, 10 µM $ZnCl_2$, 15% Glycerol, 100 µg/mL BSA, 10 mM Imidazole, 1 mM DTT, 0.5 mM PMSF, leupeptin 5µg/mL, pepstatin A 1µg/mL.

Chromatin remodeling complex or CRC buffer (2 µL): 10 mM Tris-Cl (pH 8.0), 300 mM NaCl, 1 mM $Mg(CH_3COO)_2$, 1 mM Imidazole, 2 mM EGTA, 0.1 % NP-40, 10 % Glycerol, 0.5 mM DTT, 0.5 mM PMSF, 5µg/mL leupeptin, 1µg/mL pepstatin A.

Deionized water or ATP (0.6 µL): 50 mM ATP.

Probe (2.5 µL): 10 mM Tris-Cl (pH 7.4), 100 mM NaCl, 1 mM EDTA, 5 mM DTT, 0.05% NP-40, 10 % Glycerol, 100 µg/mL BSA, 0.5 mM PMSF.

Total reaction volume: 15 µL.

Buffer used for dialysis of His-tagged Reb1 and Abf1

10 mM Hepes-KOH (pH 7.9), 300 mM NaCl, 10 µM $ZnCl_2$, 15% Glycerol, 10 mM Imidazole, 1 mM DTT, 0.5 mM PMSF.

Figure legends

Figure S1. Analyses supplementary to Figure 1. (A) Electrophoretic mobility pattern for Reb1 binding to the G-GC probe at the form of naked DNA and reconstituted nucleosome. The gel image corresponds to electrophoresis in a non-denaturing polyacrylamide gel and is representative of three independent assays. The presence of Reb1 is depicted at the top of gel images; migrations of free DNA probe (DNA), nucleosome probe (Nuc), DNA probe bound by Reb1 (Reb1-DNA) and nucleosome probe bound by Reb1 (Reb1-Nuc) are indicated at the right. **(B)** ANOVA analysis of Kd determinations for Reb1 binding site variants (Fig. 1B and Table I). **(C)** ANOVA analysis of K_{off} determinations for Reb1 binding site variants (Fig. 1C and Table I). **(D)** The complex half-life for the Reb1-T-AG probe is less than 30 seconds. *Left panel*: Schematic representation of the Reb1-T-AG binding site and the corresponding probe used in this analysis. *Right panel*: Dissociation kinetics assay. Because assays in Fig. 1C showed Reb1 dissociated from Reb1-T-AG probe in less than 5 minutes, shorter dissociation times were analyzed to determine dissociation kinetics. The image shows the three replicates performed. The time points analyzed here are depicted at the top of the gel image. Even by testing this shorter dissociation time points, no evident Reb1 binding was detected (see lanes 3-6; 9-12 and 15-18). Therefore, the dissociation constant of Reb1-T-AG probe could not be determined and the half-life of Reb1 was established to be less than 30 seconds.

Figure S2. Analyses supplementary to Figure 2. (A) Electrophoretic mobility pattern for Abf1 binding to the A_3G probe at the form of naked DNA and reconstituted nucleosome. The gel image corresponds to electrophoresis

in a non-denaturing polyacrylamide gel and is representative of three independent assays. The presence of Abf1 is depicted at the top of gel images; migrations of free DNA probe (DNA), nucleosome probe (Nuc), DNA probe bound by Abf1 (Abf1-DNA) and nucleosome probe bound by Abf1 (Abf1-Nuc) are indicated at the right. **(B)** ANOVA analysis of Kd determinations for Abf1 binding site variants (Fig. 2B and Table II). **(C)** ANOVA analysis of K_{off} determinations for Abf1 binding site variants (Fig. 2C and Table II).

Figure S3. Analyses supplementary to Figure 3A and 3B. (A) Analysis of Reb1 binding after ISW1a-mediated nucleosome sliding (supplementary to Figure 3A). **(B)** Direct measures of sliding extent and Reb1 binding percentage for T-AG and G-GC binding site variants (supplementary to Figure 3B). Bars in the graphs display the average of three independent assays for each condition analyzed (n = 3). Error bars represent one standard deviation. Asterisks denote statistically significant differences (* $p < 0.05$; ** $p < 0.01$; *** $p < 0.001$; n.s. = non-significant difference), as deducted from a two-tailed unpaired t-test.

Figure S4. Differential effect on ISW1a's sliding activity given by Reb1 and Abf1 binding site variants, determined by assays using GRF's binding removal. (A) Outline of the steps involved in the nucleosome remodeling assay. *Lower Panel*: Schematic representation depicting the remodeling pattern generated by ISW1a and the method used to quantify its activity. The "fractional sliding extent" corresponds to the ratio of intensity given by bands reflecting remodeled (slid) nucleosome over the intensity given by all bands of the nucleosome probe in the lane. **(B-C)** Nucleosome remodeling assays visualized by electrophoresis in a non-denaturing polyacrylamide gel, testing the effect of Reb1 (B) and Abf1 (C) on the sliding activity of ISW1a. The probe used in each reaction is depicted at the top of each gel picture, as well as absence or presence of ISW1a and a given GRF. Migrations of alternative forms of the nucleosome probe, which correspond to different translational positions of the histone octamer, are indicated schematically at the right of the picture. The graphs in (C) correspond to quantification of fractional sliding extent and statistical analysis. Bars in the graphs display the average of three independent assays for each condition analyzed (n = 3). Error bars represent one standard deviation. Asterisks denote statistically significant differences (*** $p < 0.001$), as deducted from a two-tailed unpaired t-test.

Figure S5 (supplementary to Figure 3D). Direct measures of sliding extent and Reb1 binding percentage for G-TC, G-GC and G-GG binding site variants. For each probe we measured ISW1a's sliding activity and binding percentage of Reb1. The graphs at the top show a comparison of ISW1a's sliding activity in presence and absence of Reb1. At the bottom, Reb1 binding percentage was measured in presence and absence of ISW1a's sliding. The probes analyzed here show that Reb1 is capable of blocking ISW1a's sliding, while remaining bound to the probes despite ISW1a's activity. For more details refer to figure 3D. Statistical significance was established by performing two-tailed unpaired Student's *t*-test (* $p < 0.05$; *** $p < 0.001$; n.s. = non-significant difference).

Figure S6 (supplementary to Figure 4). Analysis of Abf1 ability to hinder ISW1a's sliding activity, 30 minutes remodeling incubation. (A) Outline of the steps involved in the assay. **(B)** Analysis of ISW1a's sliding activity and Abf1 binding to Abf1BS variants, where ISW1a was added to the reactions after incubation with Abf1 and nucleosome sliding incubation was conducted for 30 minutes. The gel image corresponds to electrophoresis in a non-denaturing polyacrylamide gel and is representative of three independent assays. The probe used in each reaction, presence of Abf1 and ISW1a, as well as Abf1 concentrations, are depicted at the top of gel image; migrations of free DNA probe (DNA), DNA probe bound by Abf1 and nucleosome probe bound by Abf1 (Abf1-Nuc) are indicated at the right, where slid and non-slid nucleosome probe populations are represented schematically. The graphs at the right of gel image correspond to determinations of sliding extent in the presence of Abf1 relative to its absence or Abf1 binding upon ISW1a-mediated nucleosome sliding relative to its absence. All values used for these determinations were obtained from densitometric analyses of the corresponding gel scans. Bars in the graphs display the average of three independent assays for each condition analyzed. Error bars represent one standard deviation. Connectors between bars correspond to ANOVA with Tukey's multiple comparisons tests, with asterisks denoting statistically significant differences (* $p < 0.05$). **(C)** Direct measures of sliding extent and Abf1 binding percentage for Abf1 binding site variants**.** For each probe we measured ISW1a's

sliding activity and Abf1's binding. The graphs at the top show a comparison of ISW1a's sliding activity in presence and absence of Abf1. At the bottom, Abf1's binding was measured in presence and absence of ISW1a's sliding. Statistical significance was established by performing two-tailed unpaired Student's *t*-test (* $p < 0.05$; ** $p < 0.01$; *** $p < 0.001$; n.s. = non-significant difference).

Figure S7 (supplementary to Figure 4B). Direct measures of sliding extent and Abf1 binding percentage for Abf1 binding site variants, 60 minutes remodeling incubation. For each probe we measured ISW1a's sliding activity and Abf1's binding, but in these analyses the incubation period for ISW1a's sliding was doubled. The graphs at the top show a comparison of ISW1a's sliding activity in presence and absence of Abf1. At the bottom, Abf1's binding was measured in presence and absence of ISW1a's sliding. Under this longer nucleosome remodeling incubation Abf1 displayed a lower hindering of ISW1a's sliding activity, but hindering remained at similar levels comparing to 30 minutes incubation in the case of the A_3G variant (Fig. S6). Moreover, now the hindering effect given by the A_3G variant was significantly higher than hindering displayed by all the other variants (Fig. 4B). Statistical significance was established by performing two-tailed unpaired Student's *t*-test (* $p < 0.05$; ** $p < 0.01$; *** $p < 0.001$; n.s. = non-significant difference).

Figure S8 (supplementary to Figure 4C). Comparative analysis of sliding extents obtained at each time point of the kinetic analysis for Abf1. (A, B) Determinations of sliding extent in the presence of Abf1 relative to its absence at each time point (A) and Abf1 binding upon ISW1a-mediated nucleosome sliding relative to its absence at time = 0 minutes (B). All values used for these determinations were obtained from densitometric analyses of the corresponding gel scans. Bars in the graphs display the average of three independent assays for each condition analyzed. Error bars represent one standard deviation. Connectors between bars correspond to two-tailed unpaired Student's t-test, with asterisks denoting statistically significant differences (** $p < 0.01$; *** $p < 0.001$).

Figure S9. Binding strength of Reb1 and Abf1 inversely correlate with nucleosome occupancy and histone deposition in vivo. (A, B) Violin plots comparing the distribution of nucleosome occupancy (left panel) and histone deposition (right panel) levels for loci displaying low (20% bottom) and high (20% top) Reb1 (A) or Abf1 (B) occupancy, according to in vitro (PB-exo) or in vivo determinations performed by Rossi and co-workers (1). Nucleosome occupancy (histone H3) and histone deposition levels (H3-HA incorporation) were determined from genome-wide ChIP-seq data obtained by Kassem and co-workers (2). Asterisks denote statistically significant differences (** $p < 0.01$; *** $p < 0.001$; **** $p < 0.0001$), as deducted from the Mann-Whitney U test.

Figure S10. Differential sequence frequency profiles of Reb1 and Abf1 binding sites are displayed by gene bodies and promoters from ChIP-exo data. Consensus sequence exhibited by whole genome, gene bodies (ORFs) and gene promoter clusters of Reb1 (A) and Abf1 (B) binding site. The clusters were generated using ChIP-exo data (1). The logos of consensus binding sites were generated using the MEME suite (3).

Figure S11. Differential sequence frequency profiles of Reb1 and Abf1 binding sites are displayed by gene bodies and promoters from PB-exo data. Consensus sequence exhibited by gene bodies (ORFs) and gene promoter clusters of Reb1 (A) and Abf1 (B) binding site. The clusters were generated using PB-exo data (1). The PB-exo peaks were filtered by the sequence TTACCCK (Reb1) or CGTNNNNNRNGAB (Abf1) to generate the clusters. The logos of consensus binding sites were generated using the MEME suite (3).

Figure S12. Binding equilibrium for Reb1 and Abf1 is reached after 1 hour incubation. The gel images correspond to electrophoresis in a non-denaturing polyacrylamide gel. The probe used in each reaction and GRF concentrations are depicted at the top of gel images; migrations of free DNA probe (DNA), nucleosome probe (Nuc), DNA probe bound by the GRF and nucleosome probe bound by are indicated at the right. **(A)** Analyses performed for Reb1. **(B)** Analyses performed for Abf1.

Figure S13. Verification of chasing efficiency of oligonucleotides used for Reb1 and Abf1 capture in dissociation kinetics analyses. In protein-DNA dissociation assays, double stranded unlabeled competitor DNA is added after the binding reaction has proceeded, to remove the protein from its binding site and then measure the time needed for dissociation from a DNA probe. This competitor (or chaser) DNA is in large excess relative to the DNA probe and transcription factor tested, making therefore binding events between the protein and the probe negligible relative to the binding events taking place between the protein and the competitor. Any double stranded DNA sequence cannot be used as competitor DNA; the protein must preferentially bind to the chaser for efficient removal. Therefore, it is important to test the efficiency of the chaser DNAs being used before performing dissociation assays (4). **(A)** Outline of the steps involved in the assay. To test the efficiency of our chaser DNAs, we performed binding assays similar to those shown in Figs. 1B and 2B, but adding the chaser before DNA probe and GRF to the binding mix, in an 100X excess (relative to the highest GRF's final concentration in the assay: 16.6nM for Reb1 and 4.8nM for Abf1). These chaser oligos are double stranded DNA cassettes of ~20-60bp harboring Reb1 or Abf1 binding sites. The competitor DNAs being tested for Reb1 were: Reb1 T-AG and Reb1 T-CG and for Abf1 were Abf1 T-T and Abf1 A_3G (detailed sequences in table S2). The aim was to find a chaser DNA able to prevent GRF binding if added before the DNA probe. **(B)** Validation of competitor DNAs for Reb1. The image on the left shows that Reb1-T-AG is an inefficient competitor. Although the chaser oligo can prevent Reb1's binding to the Reb1-T-AG probe, it is unable to prevent binding to the Reb1-G-GG probe. The image on the right, in contrast, shows two examples of efficient Reb1 chaser DNA oligos: Reb1-G-TC-1 and Reb1-G-TC-2. Here, no Reb1 binding is seen in either of the lanes where chaser DNAs were added to the reaction. **(C)** Validation of competitor DNAs for Abf1. As shown in B, the images also display examples of inefficient (left) and efficient (right) chaser oligos, but for probes harboring Abf1 binding sites. Shown on the left is the Abf1-T-T chaser oligo, which is an efficient competitor for Abf1-T-T probe but not for Abf1-A_3G, because although little Abf1 binding can be seen, it is still significant. In contrast, the image on the right shows the Abf1-A_3G chaser oligo, which was proven to be an efficient competitor for all Abf1 binding site probes used in this paper.

Table S1. Sequence information of template plasmid and primers used for generation of each probe.

Probe name	Plasmid name and sequence harboring the region amplified in PCR reaction	Primers
NC-80/T-AG (Reb1BS)	p601-10-Reb1BS/T-AG<br>5’acaggatgtatatatctgacacgtgcctggagactagggagtaatccccttggcggttaaaacgcggg<br>ggacagcgcgtacgtgcgtttaagcggtgctagagctgtctacgaccaattgagcggcctcggcaccgg<br>gattctccagggcggccgctcgggtaaagcatcaccatggtgatgaccgcggacctgcaggcatgcaag<br>cttgagtattctatagtgtca	Forward:<br>5’/IRD700/ACAGGATGTATATAT<br>CTGACACGTGCCT<br>Reverse:<br>5’TGACACTATAGAATACTCAAGC
NC-80/G-TC (Reb1BS)	p601-10-Reb1BS/G-TC<br>5’acaggatgtatatatctgacacgtgcctggagactagggagtaatccccttggcggttaaaacgcggg<br>ggacagcgcgtacgtgcgtttaagcggtgctagagctgtctacgaccaattgagcggcctcggcaccgg<br>gattctccagggcggccggacgggtaacgcatcaccatggtgatgaccgcggacctgcaggcatgcaag<br>cttgagtattctatagtgtca	Forward:<br>5’/IRD700/ACAGGATGTATATAT<br>CTGACACGTGCCT<br>Reverse:<br>5’TGACACTATAGAATACTCAAGC
NC-80/G-GC (Reb1BS)	p601-10-Reb1BS/G-GC<br>5’acaggatgtatatatctgacacgtgcctggagactagggagtaatccccttggcggttaaaacgcggg<br>ggacagcgcgtacgtgcgtttaagcggtgctagagctgtctacgaccaattgagcggcctcggcaccgg<br>gattctccagggcggccggccgggtaacgcatcaccatggtgatgaccgcggacctgcaggcatgcaag<br>cttgagtattctatagtgtca	Forward:<br>5’/IRD700/ACAGGATGTATATAT<br>CTGACACGTGCCT<br>Reverse:<br>5’TGACACTATAGAATACTCAAGC
NC-80/G-GG (Reb1BS)	p601-10-Reb1BS/G-GG<br>5’acaggatgtatatatctgacacgtgcctggagactagggagtaatccccttggcggttaaaacgcggg<br>ggacagcgcgtacgtgcgtttaagcggtgctagagctgtctacgaccaattgagcggcctcggcaccgg<br>gattctccagggcggccgcccgggtaacgcatcaccatggtgatgaccgcggacctgcaggcatgcaag<br>cttgagtattctatagtgtca	Forward:<br>5’/IRD700/ACAGGATGTATATAT<br>CTGACACGTGCCT<br>Reverse:<br>5’TGACACTATAGAATACTCAAGC
NC-80/T-T (Abf1BS)	p601-10-Abf1BS/T-T<br>5’acaggatgtatatatctgacacgtgcctggagactagggagtaatccccttggcggttaaaacgcggg<br>ggacagcgcgtacgtgcgtttaagcggtgctagagctgtctacgaccaattgagcggcctcggcaccgg<br>gattctccagggcggccgcttatcgtattgcatgatatggtgatgaccgcggacctgcaggcatgcaagc<br>ttgagtattctatagtgtca	Forward:<br>5’/IRD700/ACAGGATGTATATAT<br>CTGACACGTGCCT<br>Reverse:<br>5’TGACACTATAGAATACTCAAGC
NC-80/A-T (Abf1BS)	p601-10-Abf1BS/A-T<br>5’acaggatgtatatatctgacacgtgcctggagactagggagtaatccccttggcggttaaaacgcggg<br>ggacagcgcgtacgtgcgtttaagcggtgctagagctgtctacgaccaattgagcggcctcggcaccgg<br>gattctccagggcggccgctaatcgtattgcatgatatggtgatgaccgcggacctgcaggcatgcaagc<br>ttgagtattctatagtgtca	Forward:<br>5’/IRD700/ACAGGATGTATATAT<br>CTGACACGTGCCT<br>Reverse:<br>5’TGACACTATAGAATACTCAAGC
NC-80/T-G (Abf1BS)	p601-10-Abf1BS/T-G<br>5’acaggatgtatatatctgacacgtgcctggagactagggagtaatccccttggcggttaaaacgcggg<br>ggacagcgcgtacgtgcgtttaagcggtgctagagctgtctacgaccaattgagcggcctcggcaccgg<br>gattctccagggcggccgcttatcgtattgcatgatagggtgatgaccgcggacctgcaggcatgcaagc<br>ttgagtattctatagtgtca	Forward:<br>5’/IRD700/ACAGGATGTATATAT<br>CTGACACGTGCCT<br>Reverse:<br>5’TGACACTATAGAATACTCAAGC
NC-80/A_3G (Abf1BS)	p601-10-Abf1BS/A_3G<br>5’acaggatgtatatatctgacacgtgcctggagactagggagtaatccccttggcggttaaaacgcggg<br>ggacagcgcgtacgtgcgtttaagcggtgctagagctgtctacgaccaattgagcggcctcggcaccgg<br>gattctccagggcggccgcttatcgtataaagtgatagggtgatgaccgcggacctgcaggcatgcaag<br>cttgagtattctatagtgtca	Forward:<br>5’/IRD700/ACAGGATGTATATAT<br>CTGACACGTGCCT<br>Reverse:<br>5’TGACACTATAGAATACTCAAGC

Letters in red correspond to the nucleosome positioning region of Widom’s 601 sequence (5).

Sequences highlighted in yellow correspond to the regions bound by the PCR primers.

Underlined sequences correspond to the binding site variants for Reb1 or Abf1 according to JASPAR database (6).

Table S2. Sequence information of oligonucleotides used in dissociation kinetics analyses.

Binding site	Forward oligonucleotide	Reverse oligonucleotide
Reb1 T-AG	5’CGGCCGC**TCGGGTAAA**GCATCAC	5’GTGATGC**TTTACCCGA**GCGGCCG
Reb1 G-TC-1	5’AATTGAGCGGCCTCGGCACCGGGATTCTCCAGGGCGGCCG G**ACGGGTAAC**GCATCAC	5’CATGGTGATGC**GTTACCCGT**CCGGCCGCCCTGGAGAATCC CGGTGCCGAGGCCGCTC
Reb1 G-TC-2	5’AATTGAGCGGCCTCGGCACCGGGATTCTCCAGGGCG**GTTA CCCGT**CTCGAGCATCAC	5’CATGGTGATGCTCGAG**ACGGGTAAC**CGCCCTGGAGAATC CCGGTGCCGAGGCCGCTC
Abf1 T-T	5’CGGCCGCTTA**TCGTATTGCATGATAT**GGTGAT	5’ATCACC**ATATCATGCAATACGA**TAAGCGGCCG
Abf1 A_3G	5’GGCCGCTTA**TCGTATAAAGTGATAG**GGTGATGACCGCGGA CCTGCA	5’GGTCCGCGGTCATCACC**CTATCACTTTATACGA**TAAGC

The double-stranded cassettes Reb1BS 1 and Abf1BS A_3G were used K_{off} and half-life determinations (Figs. 1B and 2B). Cassettes Reb1 T-AG, Reb1 G-TC-1, Reb1-G-TC-2, Abf1 T-T and Abf1BS A_3G were used in preliminary competition analysis testing the suitability of these oligonucleotides as chasers for the dissociation kinetics analyses (Fig. S13). The location of Reb1 or Abf1 binding sites, according to JASPAR database (6), is underlined.

Table S3. Position probability matrices for Reb1 binding sites obtained from ChIP-exo data, filtered by the Reb1 binding site core sequence TTACCCK.

Reb1 ChIP-exo bound sites in ORFs (filtered by TTACCCK)																	
Position	-9	-8	-7	-6	-5	-4	-3	-2	-1	0	+1	+2	+3	+4	+5	+6	+7	
A						0.273	0.000	0.000	1.000	0.000	0.000	0.000	0.000					
C						0.182	0.000	0.000	0.000	1.000	1.000	1.000	0.000					
G						0.485	0.000	0.000	0.000	0.000	0.000	0.000	0.455					
T						0.061	1.000	1.000	0.000	0.000	0.000	0.000	0.545					
Reb1 ChIP-exo bound sites in promoters (filtered by TTACCCK)																	
Position	-9	-8	-7	-6	-5	-4	-3	-2	-1	0	+1	+2	+3	+4	+5	+6	+7	
A	0.350	0.333	0.308	0.208	0.113	0.384	0.000	0.000	1.000	0.000	0.000	0.000	0.000	0.248	0.382	0.166	0.259	
C	0.166	0.168	0.180	0.238	0.257	0.134	0.000	0.000	0.000	1.000	1.000	1.000	0.000	0.248	0.365	0.272	0.151	
G	0.146	0.176	0.140	0.185	0.142	0.410	0.000	0.000	0.000	0.000	0.000	0.000	0.760	0.359	0.089	0.172	0.202	
T	0.338	0.323	0.372	0.369	0.488	0.072	1.000	1.000	0.000	0.000	0.000	0.000	0.240	0.144	0.163	0.391	0.389	

Supplementary to figure 5B, left panel

Table S4. Position probability matrices for ORFs and promoter’s loci harboring the Reb1 binding site core sequence TTACCCK.

Loci harboring TTACCCK sequence (Reb1BS core) found in ORFs																	
Position	-9	-8	-7	-6	-5	-4	-3	-2	-1	0	+1	+2	+3	+4	+5			
A					0.243	0.196	0.000	0.000	1.000	0.000	0.000	0.000	0.000	0.158	0.263			
C					0.240	0.233	0.000	0.000	0.000	1.000	1.000	1.000	0.000	0.229	0.138			
G					0.135	0.089	0.000	0.000	0.000	0.000	0.000	0.000	0.217	0.110	0.229			
T					0.382	**0.483**	1.000	1.000	0.000	0.000	0.000	0.000	**0.783**	**0.503**	**0.370**			
Loci harboring TTACCCK sequence (Reb1BS core) found in promoters																	
Position	-9	-8	-7	-6	-5	-4	-3	-2	-1	0	+1	+2	+3	+4	+5			
A	0.338	0.324	0.285	0.211	0.125	0.363	0.000	0.000	1.000	0.000	0.000	0.000	0.000	0.244	0.349			
C	0.169	0.180	0.177	0.244	0.263	0.127	0.000	0.000	0.000	1.000	1.000	1.000	0.000	0.241	**0.316**			
G	0.159	0.157	0.145	0.177	0.136	**0.316**	0.000	0.000	0.000	0.000	0.000	0.000	**0.640**	**0.317**	0.112			
T	0.334	0.339	0.392	0.368	**0.476**	0.194	1.000	1.000	0.000	0.000	0.000	0.000	0.360	0.198	0.223			

Supplementary to figure 5B, right panel. Numbers highlighted in red and bold in selected positions correspond to sequence defined as representative of its cluster at that position, considering its high frequency in the cluster and that displays the largest frequency difference between clusters. These sequence variations were used for DNA shape analysis (Figure 5C).

Table S5. Position probability matrices for Abf1 binding sites obtained from ChIP-exo data, filtered by the Abf1 binding site sequence CGTNNNNNRNGAB.

Abf1 ChIP-exo bound sites in ORFs (filtered by CGTNNNNNRNGAB)																		
Position			-7	-6	-5	-4	-3	-2	-1	0	+1	+2	+3	+4	+5	+6		
A			0.136	0.000	0.000	0.000	0.182	0.091	0.500	0.545	0.455	0.545	0.045	0.000	1.000	0.000		
C			0.455	1.000	0.000	0.000	0.136	0.273	0.136	0.227	0.091	0.000	0.364	0.000	0.000	0.318		
G			0.000	0.000	1.000	0.000	0.273	0.455	0.227	0.136	0.318	0.455	0.045	1.000	0.000	0.000		
T			0.409	0.000	0.000	1.000	0.409	0.182	0.136	0.091	0.136	0.000	0.545	0.000	0.000	0.682		
Abf1 ChIP-exo bound sites in promoters (filtered by CGTNNNNNRNGAB)																		
Position				-6	-5	-4	-3	-2	-1	0	+1	+2	+3	+4	+5	+6	+7	+8
A				0.000	0.000	0.000	0.361	0.193	0.446	0.446	0.651	0.446	0.000	0.000	1.000	0.000	0.506	0.108
C				1.000	0.000	0.000	0.217	0.325	0.169	0.120	0.169	0.000	0.145	0.000	0.000	0.349	0.108	0.253
G				0.000	1.000	0.000	0.205	0.217	0.193	0.157	0.133	0.554	0.012	1.000	0.000	0.012	0.145	0.458
T				0.000	0.000	1.000	0.217	0.265	0.193	0.277	0.048	0.000	0.843	0.000	0.000	0.639	0.241	0.181

Supplementary to figure 6B, left panel

Table S6. Position probability matrices for ORFs and promoter's loci harboring the Abf1 binding site sequence CGTNNNNNRNGAB.

Loci harboring CGTNNNNNRNGAB sequence (Abf1BS core) found in ORFs																		
Position		-8	-7	-6	-5	-4	-3	-2	-1	0	+1	+2	+3	+4	+5	+6		
A		0.346	0.344	0.000	0.000	0.000	0.195	0.282	0.279	0.259	0.307	**0.669**	0.410	0.000	1.000	0.000		
C		0.153	0.195	1.000	0.000	0.000	0.242	0.169	0.212	**0.265**	0.173	0.000	0.153	0.000	0.000	0.273		
G		0.252	0.180	0.000	1.000	0.000	0.182	0.248	0.175	0.183	**0.318**	0.331	0.212	1.000	0.000	0.280		
T		0.249	0.281	0.000	0.000	1.000	0.381	0.301	**0.333**	0.293	0.202	0.000	0.226	0.000	0.000	0.446		
Loci harboring CGTNNNNNRNGAB sequence (Abf1BS core) found in promoters																		
Position		-8	-7	-6	-5	-4	-3	-2	-1	0	+1	+2	+3	+4	+5	+6		
A		0.255	0.210	0.000	0.000	0.000	0.301	0.248	**0.439**	**0.405**	**0.486**	0.487	0.116	0.000	1.000	0.000		
C		0.188	0.262	1.000	0.000	0.000	0.193	0.216	0.154	0.121	0.196	0.000	0.210	0.000	0.000	0.272		
G		0.164	0.082	0.000	1.000	0.000	0.247	0.199	0.196	0.177	0.177	**0.513**	0.070	1.000	0.000	0.123		
T		0.393	0.446	0.000	0.000	1.000	0.259	0.336	0.211	0.297	0.142	0.000	0.603	0.000	0.000	0.605		

Supplementary to figure 6B, right panel. Numbers highlighted in red and bold in selected positions correspond to sequence defined as representative of its cluster at that position, considering its high frequency in the cluster and that displays the largest frequency difference between clusters. These sequence variations were used for DNA shape analysis (Figure 6C).

References

1. Rossi MJ, Lai WKM, Pugh BF. Genome-wide determinants of sequence-specific DNA binding of general regulatory factors. Genome Res. 2018;28(4):497-508.
2. Kassem S, Ferrari P, Hughes AL, Soudet J, Rando OJ, Strubin M. Histone exchange is associated with activator function at transcribed promoters and with repression at histone loci. Sci Adv. 2020;6(36):eabb0333.
3. Bailey TL, Johnson J, Grant CE, Noble WS. The MEME Suite. Nucleic Acids Res. 2015;43(W1):W39-49.
4. Jarmoskaite I, AlSadhan I, Vaidyanathan PP, Herschlag D. How to measure and evaluate binding affinities. Elife. 2020;9.
5. Li G, Widom J. Nucleosomes facilitate their own invasion. Nature Structural & Molecular Biology. 2004;11(8):763-9.
6. Castro-Mondragon JA, Riudavets-Puig R, Rauluseviciute I, Lemma RB, Turchi L, Blanc-Mathieu R, et al. JASPAR 2022: the 9th release of the open-access database of transcription factor binding profiles. Nucleic Acids Res. 2022;50(D1):D165-D73.

Figure S1

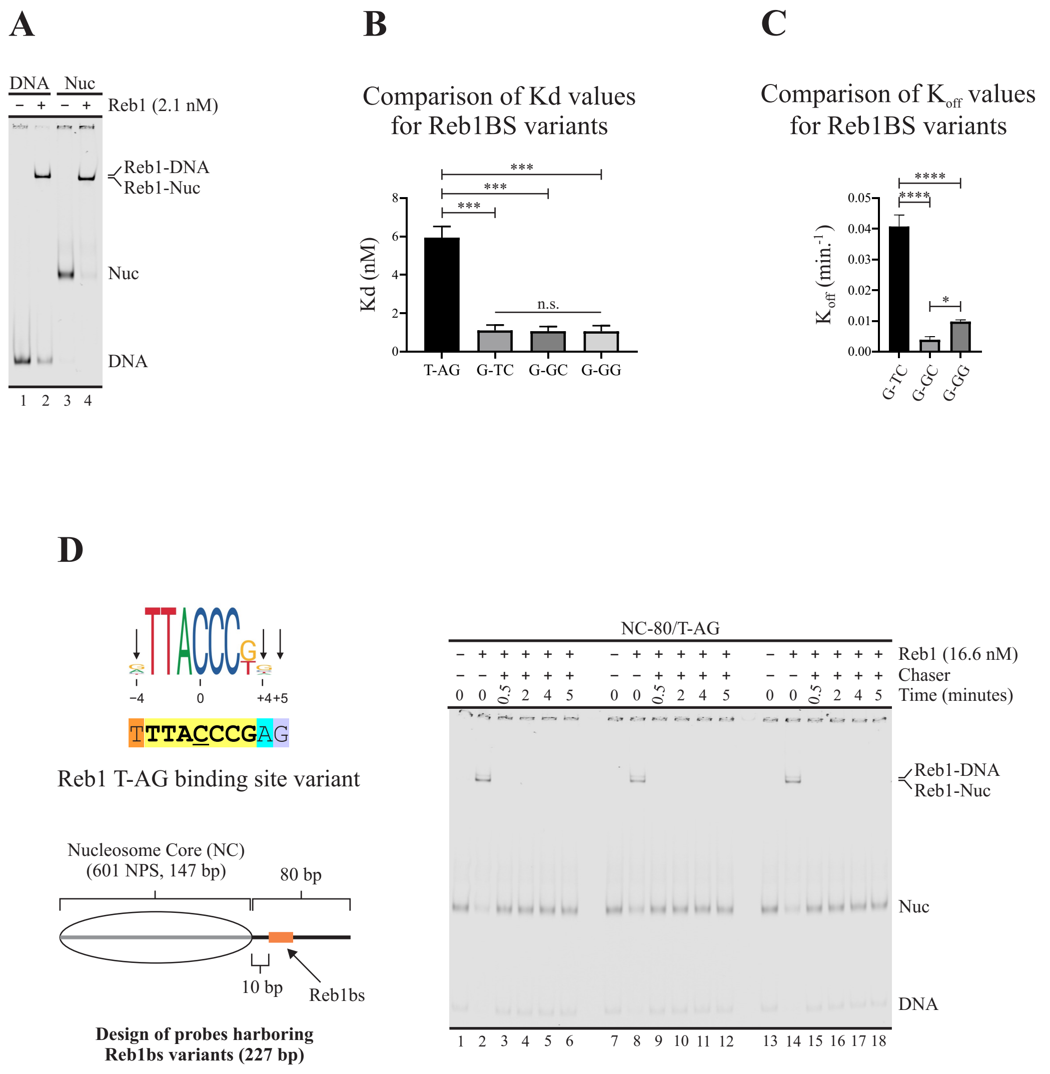
A
DNA
Nuc
Reb1 (2.1 nM)
Reb1-DNA
Reb1-Nuc
Nuc
DNA
1 2 3 4
B
Comparison of Kd values
for Reb1BS variants
Kd (nM)
***
n.s.
T-AG
G-TC
G-GC
G-GG
C
Comparison of Koff values
for Reb1BS variants
Koff (min.-1)
****
*
G-TC
G-GC
G-GG
D
-4
0
+4+5
TTTACCCGAG
Reb1 T-AG binding site variant
Nucleosome Core (NC)
(601 NPS, 147 bp)
80 bp
10 bp
Reb1bs
Design of probes harboring
Reb1bs variants (227 bp)
NC-80/T-AG
Reb1 (16.6 nM)
Chaser
Time (minutes)
Reb1-DNA
Reb1-Nuc
Nuc
DNA
1 2 3 4 5 6 7 8 9 10 11 12 13 14 15 16 17 18

Figure S2

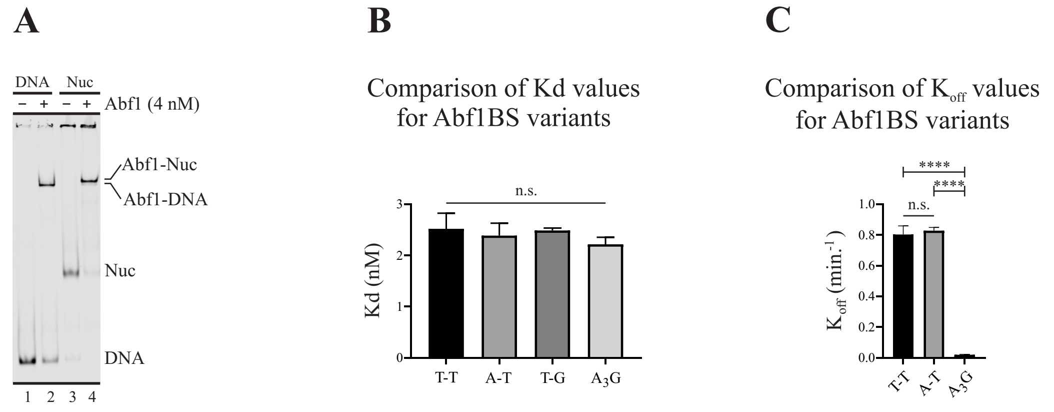

A
DNA
Nuc
Abf1 (4 nM)
Abf1-Nuc
Abf1-DNA
Nuc
DNA
1 2 3 4
B
Comparison of Kd values for Abf1BS variants
n.s.
Kd (nM)
T-T
A-T
T-G
A3G
C
Comparison of Koff values for Abf1BS variants
****
n.s.
Koff (min.-1)
T-T
A-T
A3G

Figure S3

A *(supp. to Fig.* ***3A****)*

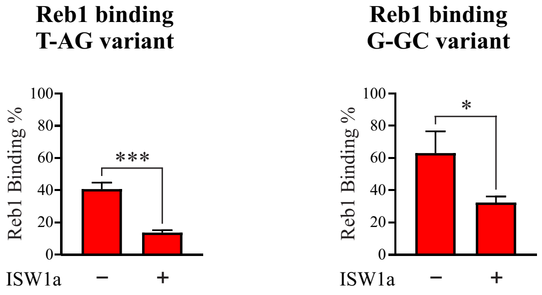

B *(supp. to Fig.* ***3B****)*

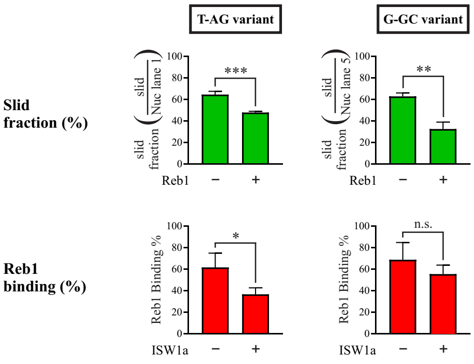

Figure S4

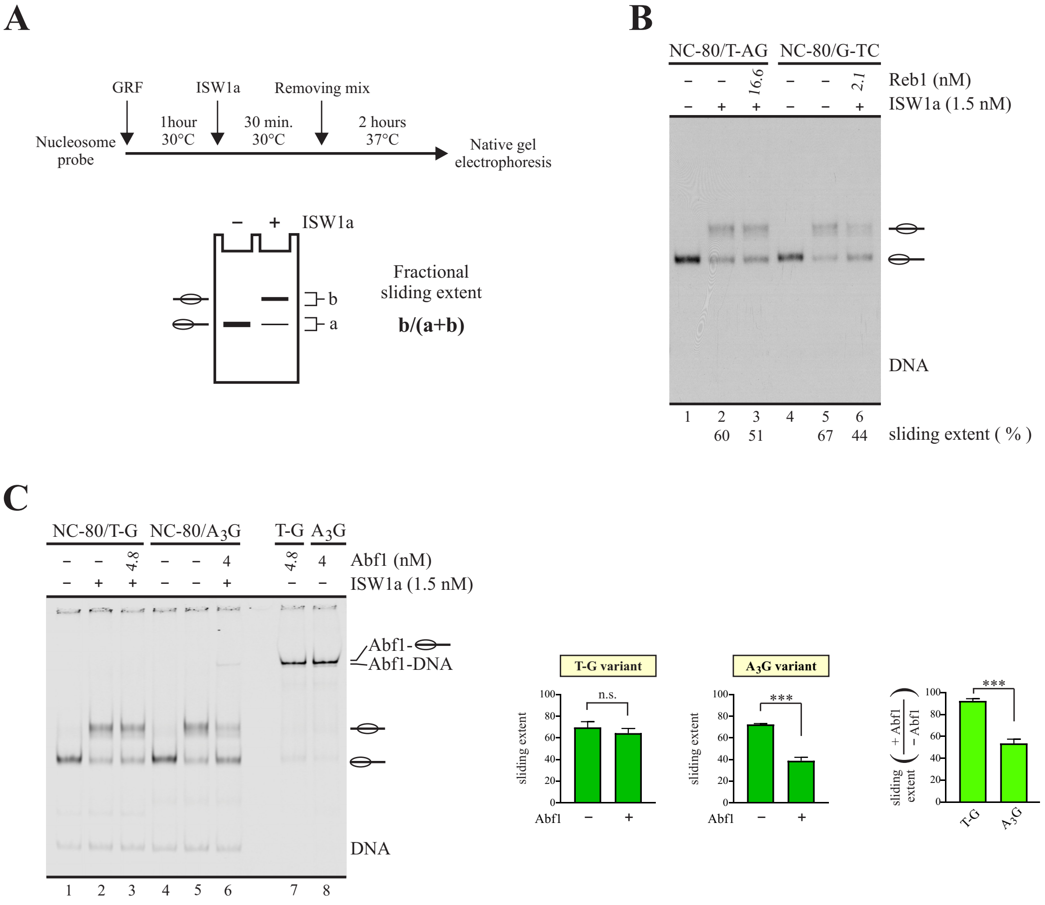
A
GRF
ISW1a
Removing mix
Nucleosome probe
1hour 30°C
30 min. 30°C
2 hours 37°C
Native gel electrophoresis
− + ISW1a
b
a
Fractional sliding extent
b/(a+b)
B
NC-80/T-AG
NC-80/G-TC
Reb1 (nM)
16.6
2.1
ISW1a (1.5 nM)
DNA
1 2 3 4 5 6
60 51 67 44
sliding extent (%)
C
NC-80/T-G
NC-80/A3G
T-G
A3G
Abf1 (nM)
4.8
4
ISW1a (1.5 nM)
Abf1-
Abf1-DNA
DNA
1 2 3 4 5 6 7 8
T-G variant
A3G variant
n.s.
***
sliding extent
Abf1
+ Abf1
− Abf1
T-G
A3G

Figure S5

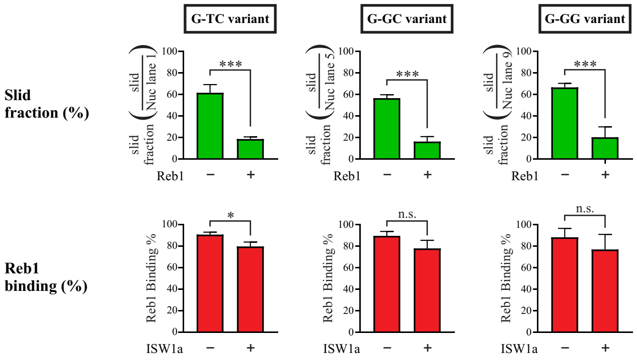

G-TC variant
G-GC variant
G-GG variant
Slid fraction (%)
Reb1 binding (%)
slid fraction (slid / Nuc lane 1)
slid fraction (slid / Nuc lane 5)
slid fraction (slid / Nuc lane 9)
***
*
n.s.
Reb1 Binding %
Reb1
ISW1a
–
+
0
20
40
60
80
100

Figure S6

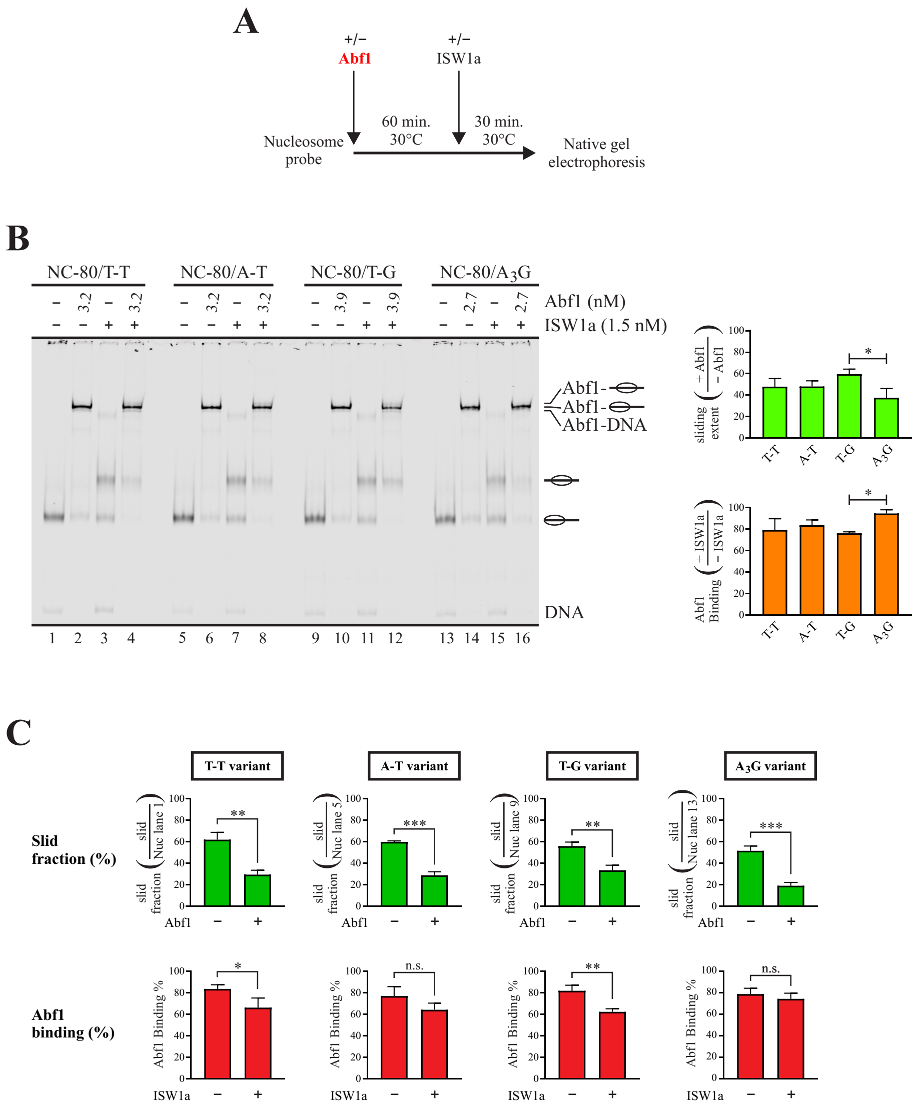
A
+/−
Abf1
+/−
ISW1a
60 min.
30°C
30 min.
30°C
Nucleosome probe
Native gel electrophoresis
B
NC-80/T-T
NC-80/A-T
NC-80/T-G
NC-80/A3G
Abf1 (nM)
ISW1a (1.5 nM)
Abf1-
Abf1-
Abf1-DNA
DNA
1 2 3 4 5 6 7 8 9 10 11 12 13 14 15 16
sliding extent
Abf1 Binding
T-T
A-T
T-G
A3G
C
T-T variant
A-T variant
T-G variant
A3G variant
Slid fraction (%)
Abf1 binding (%)
slid fraction
Abf1 Binding %
Abf1
ISW1a
n.s.

Figure S7

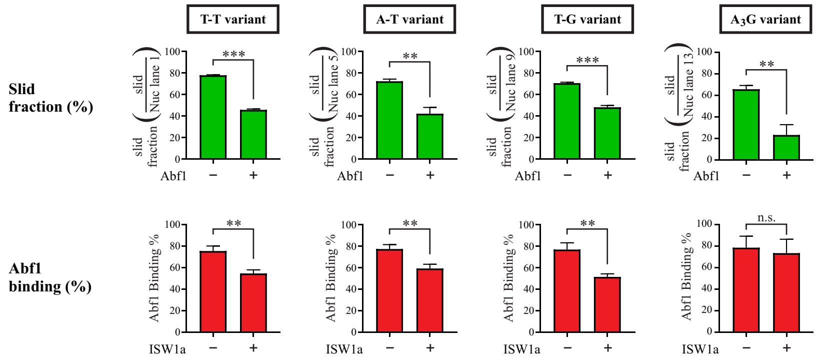

T-T variant
A-T variant
T-G variant
A$_3$G variant
Slid fraction (%)
Abf1 binding (%)
slid fraction (slid / Nuc lane 1)
slid fraction (slid / Nuc lane 5)
slid fraction (slid / Nuc lane 9)
slid fraction (slid / Nuc lane 13)
Abf1 Binding %
Abf1
ISW1a
***
**
n.s.

Figure S8

A

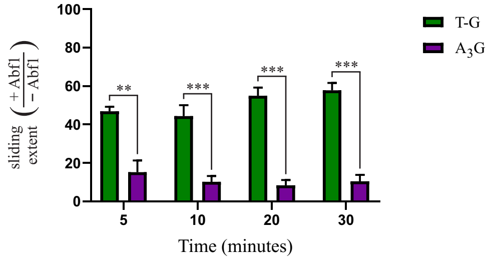

100
80
60
40
20
0
sliding extent (+ Abf1 / − Abf1)
**
***
***
***
5
10
20
30
Time (minutes)
T-G
A_3G

B

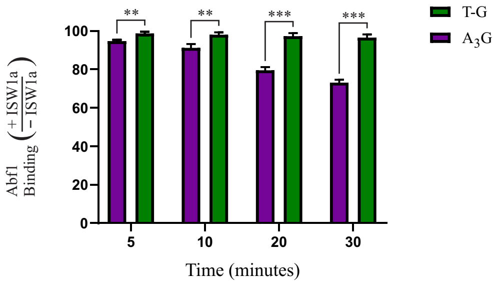

**
**
***
***
100
80
60
40
20
0
Abf1 Binding (+ ISW1a / − ISW1a)
5
10
20
30
Time (minutes)
T-G
A_3G

Figure S9

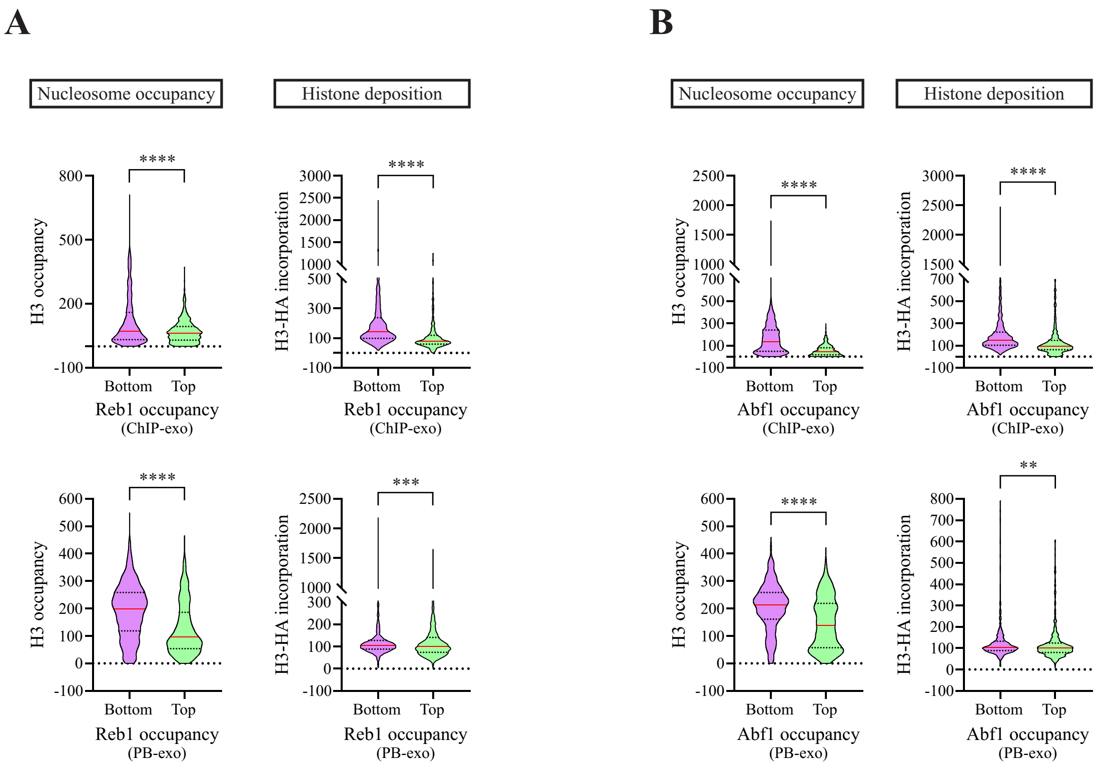
A
B
Nucleosome occupancy
Histone deposition
H3 occupancy
H3-HA incorporation
****
***
**
Bottom
Top
Reb1 occupancy
(ChIP-exo)
(PB-exo)
Abf1 occupancy

Figure S10

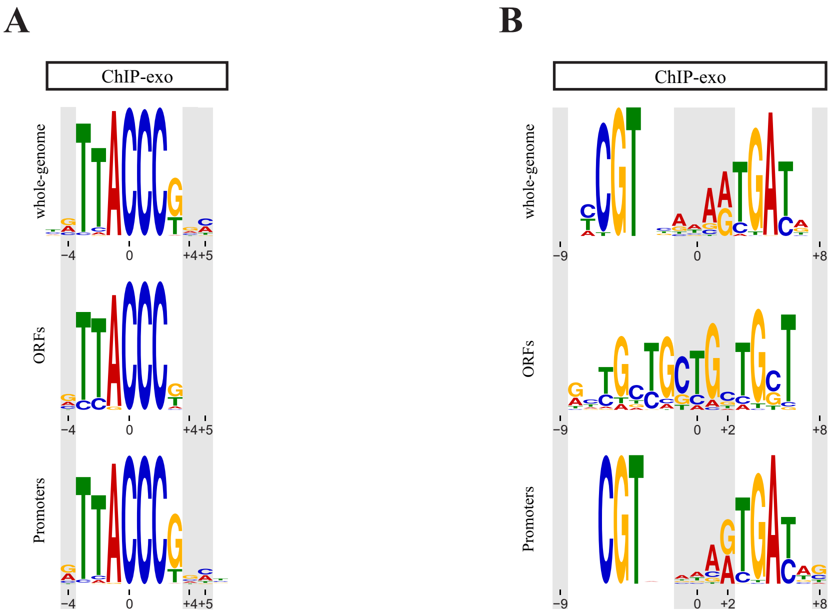

A
B
ChIP-exo
ChIP-exo
whole-genome
ORFs
Promoters
whole-genome
ORFs
Promoters
−4
0
+4+5
−9
0
+2
+8

Figure S11

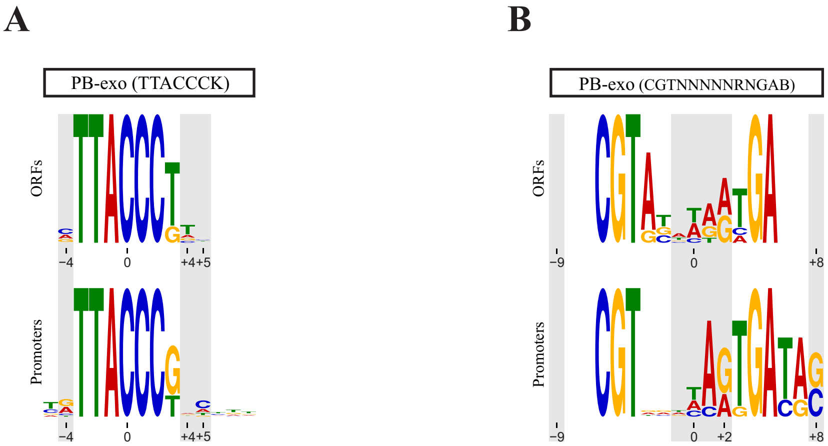
A
B
PB-exo (TTACCCK)
PB-exo (CGTNNNNNRNGAB)
ORFs
Promoters
−4
0
+4+5
−9
0
+2
+8

Figure S12

A

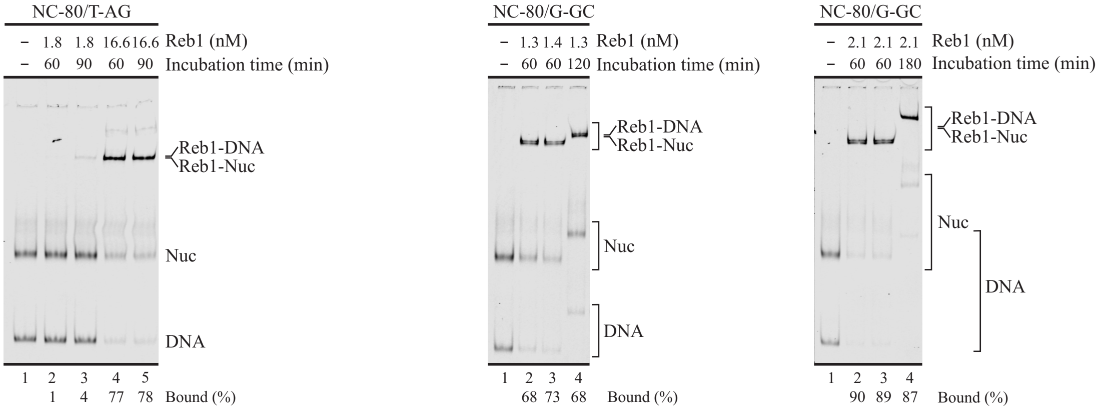

B

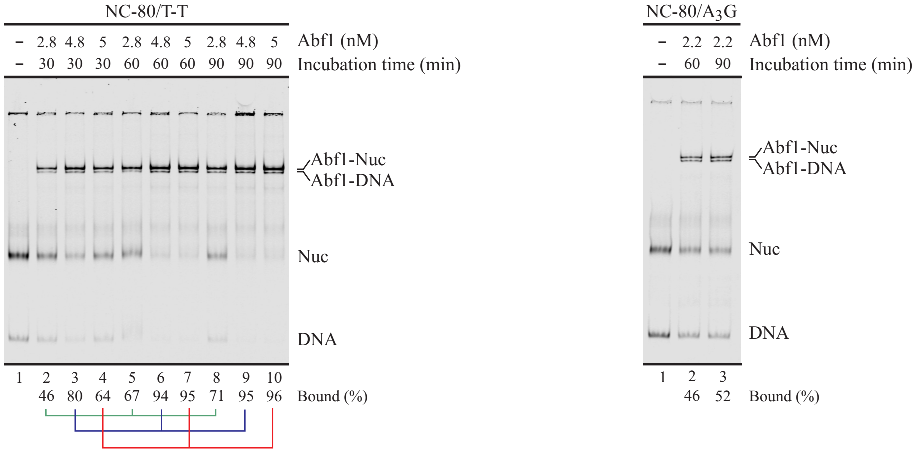

Figure S13

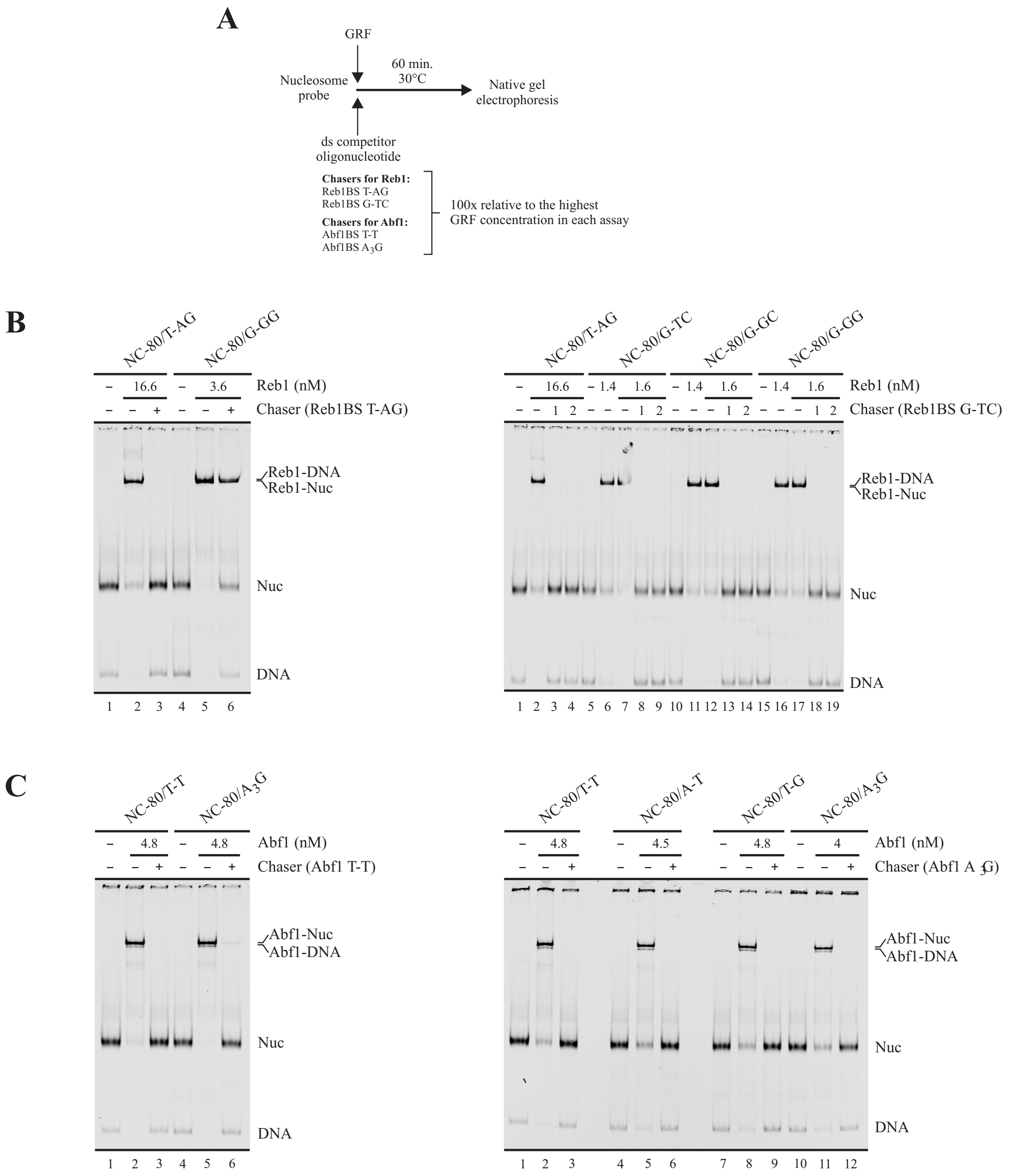
A
GRF
Nucleosome probe
60 min. 30°C
Native gel electrophoresis
ds competitor oligonucleotide
Chasers for Reb1:
Reb1BS T-AG
Reb1BS G-TC
Chasers for Abf1:
Abf1BS T-T
Abf1BS A3G
100x relative to the highest GRF concentration in each assay
B
NC-80/T-AG
NC-80/G-GG
Reb1 (nM)
16.6
3.6
Chaser (Reb1BS T-AG)
Reb1-DNA
Reb1-Nuc
Nuc
DNA
1 2 3 4 5 6
NC-80/T-AG
NC-80/G-TC
NC-80/G-GC
NC-80/G-GG
Reb1 (nM)
16.6
1.4
1.6
Chaser (Reb1BS G-TC)
Reb1-DNA
Reb1-Nuc
Nuc
DNA
1 2 3 4 5 6 7 8 9 10 11 12 13 14 15 16 17 18 19
C
NC-80/T-T
NC-80/A3G
Abf1 (nM)
4.8
Chaser (Abf1 T-T)
Abf1-Nuc
Abf1-DNA
Nuc
DNA
1 2 3 4 5 6
NC-80/T-T
NC-80/A-T
NC-80/T-G
NC-80/A3G
Abf1 (nM)
4.8
4.5
4
Chaser (Abf1 A3G)
Abf1-Nuc
Abf1-DNA
Nuc
DNA
1 2 3 4 5 6 7 8 9 10 11 12
